# Simulating Molecular Mechanisms of the MDM2-Mediated Regulatory Interactions: A Conformational Selection Model of the MDM2 Lid Dynamics

**DOI:** 10.1371/journal.pone.0040897

**Published:** 2012-07-16

**Authors:** Gennady M. Verkhivker

**Affiliations:** 1 School of Computational Sciences and Crean School of Health and Life Sciences, Schmid College of Science and Technology, Chapman University, Orange, California, United States of America; 2 Department of Pharmacology, University of California San Diego, La Jolla, California, United States of America; University of Leeds, United Kingdom

## Abstract

Diversity and complexity of MDM2 mechanisms govern its principal function as the cellular antagonist of the p53 tumor suppressor. Structural and biophysical studies have demonstrated that MDM2 binding could be regulated by the dynamics of a pseudo-substrate lid motif. However, these experiments and subsequent computational studies have produced conflicting mechanistic models of MDM2 function and dynamics. We propose a unifying conformational selection model that can reconcile experimental findings and reveal a fundamental role of the lid as a dynamic regulator of MDM2-mediated binding. In this work, structure, dynamics and energetics of apo-MDM2 are studied as a function of posttranslational modifications and length of the lid. We found that the dynamic equilibrium between “closed” and “semi-closed” lid forms may be a fundamental characteristic of MDM2 regulatory interactions, which can be modulated by phosphorylation, phosphomimetic mutation as well as by the lid size. Our results revealed that these factors may regulate p53-MDM2 binding by fine-tuning the thermodynamic equilibrium between preexisting conformational states of apo-MDM2. In agreement with NMR studies, the effect of phosphorylation on MDM2 interactions was more pronounced with the truncated lid variant that favored the thermodynamically dominant closed form. The phosphomimetic mutation S17D may alter the lid dynamics by shifting the thermodynamic equilibrium towards the ensemble of “semi-closed” conformations. The dominant “semi-closed” lid form and weakened dependence on the phosphorylation seen in simulations with the complete lid can provide a rationale for binding of small p53-based mimetics and inhibitors without a direct competition with the lid dynamics. The results suggested that a conformational selection model of preexisting MDM2 states may provide a robust theoretical framework for understanding MDM2 dynamics. Probing biological functions and mechanisms of MDM2 regulation would require further integration of computational and experimental studies and may help to guide drug design of novel anti-cancer therapeutics.

## Introduction

The p53 tumor suppressor is known as “The Guardian of the Genome” [1] and plays a fundamental role in maintaining the integrity of the genome by inducing either cell cycle arrest or apoptosis following cellular stress signals [2], [3]. In normal cells the level of p53 is tightly regulated and maintained at a low level by the murine double minute (MDM2) oncoprotein, which is a p53-specific E3 ubiquitin ligase [4]–[6]. MDM2 recognizes the N-terminal trans-activation domain of the p53 tumor suppressor and is responsible for inactivation of p53 transcription and targeting p53 for ubiquitin-mediated degradation [7]–[9]. MDM2 can inhibit p53 activity by nucleo-cytoplasmic shuttling [10], [11] or by binding of its N-terminal domain to the trans-activation domain of p53 [12], [13]. Overexpression of the MDM2 oncogene is common in a variety of malignant human tumors and contributes to inactivation of p53 [14]–[25]. MDM2 consists of several conserved domains including the N-terminal domain that binds the α-helix from the N- terminal transactivation domain of p53 [10]–[12]. The N-terminal MDM2 domain is followed by the nuclear localization signal (NLS) and nuclear export signal (NES). Acidic domain occupies the central region, the zinc finger (ZF) domain and the RING finger (RF) domains are at the C-terminus [11]. Genotoxic stress and DNA damage [26]–[28] can cause multiple post-translational modifications, which are spread across different domains in both p53 and MDM2 [29]–[38], and allow p53 to escape MDM2 inhibition and degradation. Although phosphorylation of the conserved residues S15 and S20 at the amino terminus of p53 could decrease association with MDM2 [39], [40], this may not be a universal requirement for stabilization of p53 [41]. The phosphorylation sites T18 and S20 at the p53-MDM2 interface could also modulate binding to MDM2, thus upregulating p53 levels in stressed cells [42]. However, biochemical experiments have suggested that phosphorylation of p53 sites alone may not be sufficient to disrupt the p53-MDM2 interactions and synergistic coupling of p53 and MDM2 phosphorylatable sites may be required for regulation of p53 activity [43], [44]. Among functionally important phosphorylation sites at the N-terminal MDM2 domain is the S17 residue that can be phosphorylated by DNA-dependent protein kinase *in vitro* [26]. The MDM2 phosphorylation sites are located not only at the N-terminal domain, but also within the central domain, and near the carboxyl terminal of the RING domain [29]–[38]. Following DNA damage, phosphorylation of MDM2 sites could lead to the concomitant changes in the protein function and stabilization of p53. To protect p53, several signaling pathways induced by genotoxic stress could alter the ability of MDM2 to neutralize p53 through inhibition of the MDM2-mediated ubiquitination of p53 [29]–[38].

Structural basis of the MDM2-mediated p53 regulation was first addressed based on the crystal structure of the N-terminal domain of MDM2 bound to p53, revealing a deep hydrophobic cleft in MDM2 to which the p53 peptide binds as an amphipathic α-helix [45]. This study has supported the hypothesis that MDM2 can inactivate the tumor suppressor by shielding the transactivation domain of p53 from transcriptional machinery [45]–[48]. The functional effects of p53 phosphorylation sites and peptide length on p53-MDM2 regulation, initially addressed in a series of early biochemical investigations [49]–[51], were further detailed by using fluorescence anisotropy competition assay [52]. Although posttranslational modifications of Ser-15 and Ser-20 did not affect p53 binding to MDM2, phosphorylation of Thr-18 resulted in **∼**20-fold binding reduction in binding, suggesting a plausible regulatory scenario for disruption of p53-MDM2 binding. In addition, this study has evidenced the tighter binding of a smaller peptide, p53 (18−26) (*K*_d_ **∼**70 nM), where the additional truncation to p53 (19−26) resulted in a 10-fold reduction in affinity [52]. Structure-functional studies of MDM2 binding with p53-based peptides have revealed significant variations in binding affinities depending on the peptide length that can be accompanied by local and global conformational changes in the MDM2 receptor [53], [54]. These studies have for the first time demonstrated that ligand-based modulation of the receptor dynamics is an important organizing principle of MDM2 function. NMR studies of the p53-derived peptides binding with MDM2 have confirmed the important role of Thr-18 by showing that the p53-truncated peptide (17–26) can increase binding affinity by 13-fold, while the deletion of Thr-18 in a shorter p53 peptide variant (19–26) completely abolished binding [53]. This NMR study has also suggested that peptide binding may elicit global conformational changes of MDM2, spreading beyond local adjustments in the binding cleft [53]. Subsequent NMR spectroscopy study has confirmed that peptide-induced structural changes in MDM2 and binding affinities of p53-derived peptides can depend on the peptide length [54]. Indeed, the isothermal titration calorimetry experiments have demonstrated a significant improvement in MDM2 biding between p53 (15–29) (*K*_d_ ∼500 nM) and p53 (17–26) (*K*_d_ ∼50 nM), reflecting the key role of enthalpy-entropy compensation in driving binding of p53-MDM2 complexes [54]. Overall, the enhanced binding of smaller p53-based peptides, that include Thr-18 and terminate at Leu-26, emerged as important evidence guiding subsequent structure-functional studies of p53-MDM2 regulation and design of p53-based peptidomimetics.

The pioneering NMR study of apo-MDM2 (residues 16–125) has discovered that the stretch of the N-terminal MDM2 residues (residues 16–24) can form a flexible lid competing for the p53 binding site on MDM2 via a pseudo-substrate mechanism [55]. This has led to the initial conjecture of p53 regulation by posttranslational MDM2 modifications. According to this model, under normal conditions the lid would weakly interact with the binding cleft and can be readily displaced by p53, whereas phosphorylation of the S17 lid residue may induce a stable lid conformation, “closing” over the p53 binding cleft and thus inhibiting the p53-MDM2 interactions [55]. This study has proposed that rapid in-vivo phosphorylation of the “structural neighbors” p53-T18, p53-S20 and MDM2-S17 may potentially induce the close proximity of the phosphate groups on these residues, thus leading to the eventual abrogation of the p53-MDM2 interactions and activation of p53 [55]. The complete N-terminal lid construct (residues 1–24) was not resolved in the crystal structure of the p53-MDM2 complex [45], neither it was well-defined in the NMR ensemble of apo-MDM2 structures [56]. Nevertheless, both NMR investigations [55], [56] have consistently observed a partial ordering of the lid motif 18-QIPASEQ-24 that can form meta-stable intramolecular interactions in the binding site. According to the proposed hypothesis, the closed form of the MDM2 lid may be compatible with the smaller p53-based peptide (17–26), thus explaining the enhanced binding affinity of p53 analogues terminating at Leu-26 [56]. Multidimensional solution NMR studies of the N-terminal domain of human MDM2 (residues 17–125) have provided the first comprehensive analysis of the protein dynamics in the apo-MDM2 state, the p53-bound state, and in the complex with the nutlin-3 inhibitor [57]. This study has shown that the lid can predominantly exist in the “closed” form that can effectively shield MDM2 from binding of large peptides, yet the lid can be displaced by the p53(17–29) peptide. Functional dynamics of the MDM2 lid has revealed a slow exchange (>10-ms time scale) between a dominant “closed” form and a minor disordered form, corresponding to the p53-bound MDM2 state. It was also discovered that high-affinity p53-based peptides terminating at Leu-26 and small molecule inhibitors, such as nutlin-3, can be structurally compatible with the closed lid form [57]. The crystal structures [58], [59] and NMR structures [60] of the MDM2 complexes with active cis-imidazoline analogs (also known as nutlins) have unveiled that these inhibitors can utilize the imidazole ring as a scaffold to mimic the p53-MDM2 interactions by projecting its three hydrophobic groups into the respective hydrophobic pockets of the MDM2 binding cleft. However, none of these early experiments has included the lid motif in the structure determination, and thus the specific regulatory role of the MDM2 lid in accommodating ligand binding could not be initially tested.

The complexity of MDM2-mediated regulatory mechanisms and challenges associated with the rationalization of posttranslational modification effects was evidenced from conflicting results obtained in NMR [55], [57] and enzymological studies [61], [62]. NMR studies have suggested that phosphorylation at S17 and phosphomimetic mutation S17D can induce a “closed” form of the lid, thus shielding the MDM2 binding cleft from p53 and inhibiting the p53-MDM2 complex formation [55], [57]. Recent biochemical studies challenged this mechanism by offering a contrasting mechanistic hypothesis of the MDM2-mediated regulation [61], [62]. According to this alternative model, phosphorylation and mutations at S17 would alter the lid dynamics and result in the opening of the hydrophobic cleft, thus promoting formation of the p53-MDM2 complex [61], [62]. The supporting evidence was based on biochemical studies of MDM2 mutants, where the phosphomimetic mutation S17D was shown to increase the stability of the p53-binding domain of MDM2 and facilitate MDM2-mediated ubiquitination of p53 [61]. It was also shown that the phosphomimetic lid could increase the thermostability of the MDM2 protein in the presence of nutlins, p53-mimetic peptides and other small molecule inhibitors [62]. Mutagenesis experiments from these studies have revealed that R97S and K98P mutants do not structurally perturb the MDM2 domain, yet the enhanced binding of S17D to p53 can be disrupted in the S17D/R97S/K98P triple mutant. Based on these indications, it was suggested that chemical modifications at S17 may induce opening of the binding cleft by promoting migration of the lid from the binding site and the formation of stabilizing interactions with the surface residues R97 and K98 [61], [62]. However, this alternative model could not reconcile the contradictory findings where the phosphomimetic lid should enhance the thermostability of MDM2 in the presence of p53-mimetic peptides (according to the enzymological experiments [61], [62], yet ligand binding is thermodynamically favored by the “closed” lid form of the MDM2 receptor (according to NMR [55], [57]). The latest chapter in the ongoing debates about the role of MDM2 phosphorylation in p53 activation was presented in an illuminating comparative analysis of the p53-binding domain MDM2 (1−109), Ser17-phosphorylated analogue pS17 and S17D [63]. Structure and binding thermodynamics of these complete MDM2 variants were probed by a panel of p53-derived peptide ligands using a combination of complementary experimental approaches, including surface plasmon resonance, fluorescence polarization, NMR and CD spectroscopic techniques [63]. This extensive biophysical characterization of MDM2 proteins has for the first time demonstrated that the complete N-terminal lid construct (residues 1−24) can adopt a partially structured, closed conformation in apo-MDM2 that can weaken binding of p53-derived peptides in a size-dependent manner. The results of this comprehensive study were largely consistent with the earlier NMR studies [55], [57] in supporting the “closed” model of the lid occluding the p53-binding site on MDM2. Although NMR and CD analyses have confirmed the presence of stabilizing intramolecular interactions mediated by pS17 and S17D, these energetic effects were found to be transient in nature and rather weak [63]. The central and somewhat surprising finding of this study suggested that although phosphorylation at S17 may be functionally important to stabilize the closed form of the lid, binding thermodynamics of p53-based peptides can be largely independent on the phosphorylation state of the lid at structural and functional levels [63]. These studies have thus highlighted that the mechanism of lid-induced regulation of p53 binding may be modulated not only by the phosphorylation state, but also by the length of the lid construct. Indeed, phosphorylation of the truncated lid (residues 16–24) has resulted in a significant functional effect on p53 binding seen in NMR studies [55], [57] as opposed to a considerably weaker effect observed in biophysical studies with the complete lid (residues 1–24) [63]. The crystallographic and NMR studies have similarly offered conflicting observations regarding ligand-induced conformational changes in MDM2. The solution structure of apo-MDM2 (residues 2–118) [56] has suggested that considerable conformational changes may accompany p53 binding, thus leading to a more open and expanded MDM2 structure in complexes with small molecules and peptides. However, the recent NMR studies have convincingly demonstrated that the overall structure and dynamics of apo-MDM2 and the peptide-bound MDM2 complexes is similar and conformational changes modulated by ligand binding may be primarily localized in the MDM2 lid region and in the portions of the binding cleft [55], [57]. NMR-based investigation of MDM2 phosphorylation effects on p53 activation has also demonstrated that posttranslational modifications do not induce significant conformational changes in the apo protein [63]. Furthermore, the crystal structures of the MDM2 complexes with peptidomimetics [64]–[75] and small molecule inhibitors [58]–[60], [76]–[80] appeared to be quite similar, suggesting that ligand binding would not necessarily elicit global conformational changes of the MDM2 receptor.

Computational studies of p53-MDM2 binding were pioneered by late Peter Kollman and coworkers when they introduced a computational alanine scanning approach to evaluate contributions of the p53 residues to the binding free energy [81]. This approach has been further developed to examine the p53-MDM2 interactions and evaluate the role of key residues in the human p53-MDM2 complex for computational design of β-peptide p53-mimetics [82]. Pharmacophore-based computational modeling was successfully applied to design libraries of p53 mimics [83]. Computational design of p53-derived peptides has proposed that peptide extensions may enhance binding affinities by modulating the interactions beyond the binding cleft [84]. Molecular dynamics (MD) simulations have elucidated conformational changes in the p53-binding cleft suggesting that a wider and more stable topology of the binding cleft may be induced in the MDM2 complexes, while apo-MDM2 could favor a narrower and more flexible binding site [85], [86]. Computer simulations of the p53 transactivation domain have shown that phosphorylation at T18 and S20 residues would not disrupt the helical structure of p53, but could rather reduce the p53-MDM2 binding affinities [87]. Subsequent studies have shown that p53 binding to MDM2 could be modulated by phosphorylation at T18, whereby the affinity of the phosphorylated p53 peptide may be enhanced by compensatory mutations of the Y67, D68 and E69 residues [88]. In addition, thermodynamics and kinetics of p53-MDM2 binding can be controlled by functional dynamics of the gate-keeper MDM2 residue Y100 [89]. The effect of chemical modifications in the lid on p53-MDM2 binding was initially addressed using MD simulations of the N-terminal apo-MDM2 domain [90]. This study has for the first time attempted to model the complex relationship between the MDM2-mediated regulation of p53 binding, the MDM2 lid dynamics and a chemical state of phosphorylatable residues. Nevertheless, this investigation could not yield an intrinsically consistent thermodynamic model that simultaneously satisfied conflicting observations from NMR [55], [57] and biochemical studies [61], [62]. Biophysical modeling studies have successfully reproduced the experimental trends in binding affinities with MDM2 for a large variety of p53 peptides [91]. MD simulations have been also used to rationalize the differences in binding of p53 and nutlin-3 to the MDM2 and MDMX receptors, revealing that p53 may be displaced from MDM2 by nutlin-3 because of the larger entropic penalty upon sequestration from MDM2 [92]. Molecular docking with multiple MDM2 crystal structures using a receptor-based pharmacophore model has identified binding hotspots and assisted in the discovery of high-affinity small-molecule inhibitors [93]. MD simulations combined with molecular mechanics generalized Born surface area (MM-GBSA) analysis have successfully predicted the relative MDM2 binding affinities for a number of p53 analogues, including regulatory phosphorylation, p53 mutations and truncations [94]–[102]. Mechanistic and thermodynamic effects of C-terminal MDM2 mutations have revealed distinct mechanisms for modulating binding affinity that could guide the design of targeted peptidomimetics and small molecules [103]. Overall, integration of computational and experimental approaches has become a viable strategy in the discovery of MDM2 inhibitors targeting p53-MDM2 binding [104], [105].

Despite the growing body of structural and functional experiments, mechanistic and dynamic aspects of MDM2-mediated regulatory mechanisms remain rather confusing, owing in part to conflicting data produced in NMR [55], [57] biochemical [61], [62] and biophysical studies [63]. Particularly intriguing recent evidence has indicated that structural and functional impact of posttranslational lid modifications may be affected not only by the length of the p53-based peptides, but be also influenced by the lid construct and its size [63]. In this work, we propose a unifying theoretical model capable of reconciling structural, biophysical and computational studies. An integrative computational analysis is employed to simulate structure, dynamics and energetics of apo-MDM2 as a function of posttranslational modifications. Similarities and differences in functional dynamics of apo-MDM2 with the complete and truncated lids are also analyzed. We show that a conformational selection model of preexisting MDM2 states can provide a unifying mechanistic principle of MDM2 dynamics and reveal an important functional role of the lid in dynamic regulation of MDM2 functions.

## Results

### MD Simulations of apo-MDM2

**Figure 1:**
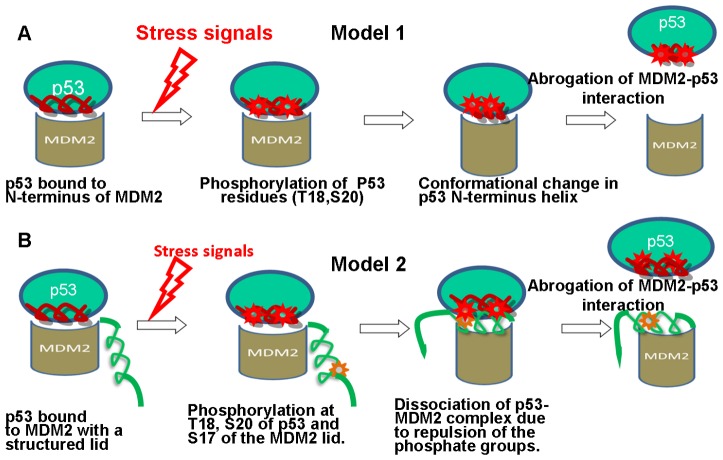
Models of the MDM2-mediated Regulation Mechanisms. (**A**) The first model assumed a conformational change in the N-terminus amphipathic helix of p53 upon phosphorylation of T18. According to this model, conformational changes in p53 could reduce binding affinity and result in the eventual disruption of the p53-MDM2 interactions. (**B**) The second model suggested the role of phosphorylation at S17 in displacing the phosphorylated p53 from the binding site. A simultaneous in-vivo phosphorylation of T18 and S20 on p53 and S17 on the MDM2 lid can bring the negatively charged phosphates on these residues in a close proximity leading to the abrogation of the p53-MDM2 interactions.

Several models were initially proposed to explain mechanisms of the p53-MDM2 interactions and regulation of p53 (**Figure 1**). The first model invoked a conformational change in the interacting p53-helix upon phosphorylation of p53-T18 which could lead to the reduced affinity for MDM2 [49]–[51]. The alternative model suggested the role of the phosphorylated residue S17 as a functional regulator of MDM2 interactions by inducing a “closed” lid conformation which is capable of competing with p53 for binding with MDM2 [55]. According to this model, rapid phosphorylation of the MDM2-S17 residue, followed by phosphorylation of p53-T18, would make the displacement of the MDM2 lid difficult due to a close proximity of the phosphate groups and result in the eventual repulsion of p53. To elucidate structural and dynamic aspects of the MDM2-mediated regulation of p53, we employed a synergistic modeling approach that combined all-atom MD simulations and analysis of the equilibrium conformational ensembles with molecular docking, structural clustering and binding free energy analyses of the low-energy structures. MD simulations of the N-terminal domain of apo-MDM2 using a truncated functional lid (residues 16-TSQIPASEQ-24) [55], [57] and a complete lid (residues 1–24) [56], [63] were carried out for the wild type (WT) form, the phosphorylated form MDM2-pS17, and the phosphomimetic form MDM2-S17D. The following specific objectives were pursued in MD simulations : (a) perform a comparative analysis of the equilibrium conformational ensembles using both complete and truncated lid representations; (b) elucidate the role of phosphorylation and phosphomimetic mutation on the mechanism of MDM2 regulation; (c) quantify the effect of the lid size ((16−125)MDM2 [55], (17−125)MDM2 [57], (1–125) MDM2 [63]) on functional dynamics and the intramolecular regulatory interactions; (d) identify key functional residues and interactions that control dynamics and regulation of MDM2 binding.

**Figure 2:**
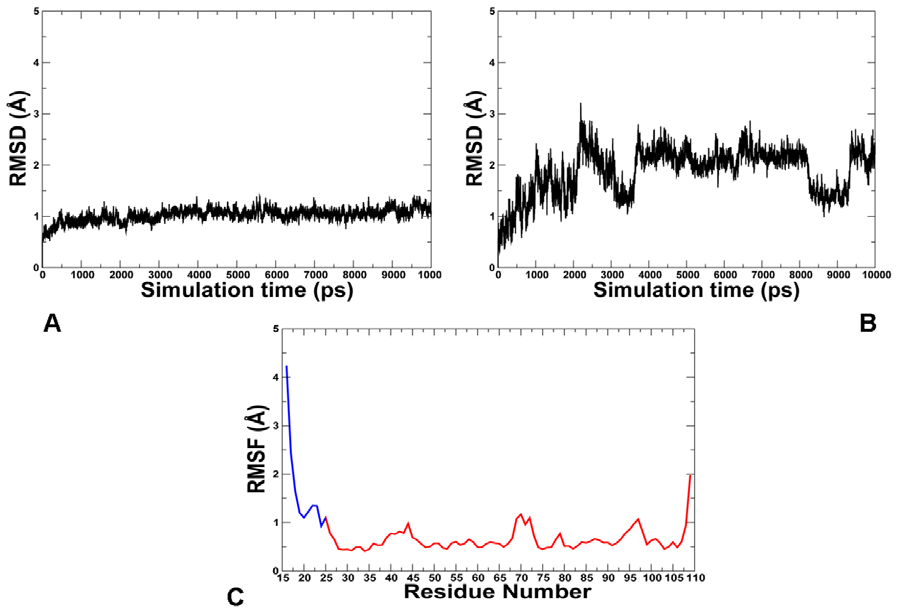
MD Simulations of the Phosphorylated pS17 MDM2 form. MD simulations were carried out using a truncated lid form (residues 16–24). The RMSD fluctuations of the Cα atoms of the core MDM2 domain residues 25–109 (**A**) and the MDM2 lid residues 16–24 (**B**) obtained from 10 ns MD simulations of the phosphorylated pS17 form. (**C**) The RMSF values of the Cα atoms of the core MDM2 domain residues 25–109 (shown in red) and the MDM2 lid residues 16–24 (shown in blue) obtained from 10 ns MD simulations of the phosphorylated pS17 form.

**Figure 3:**
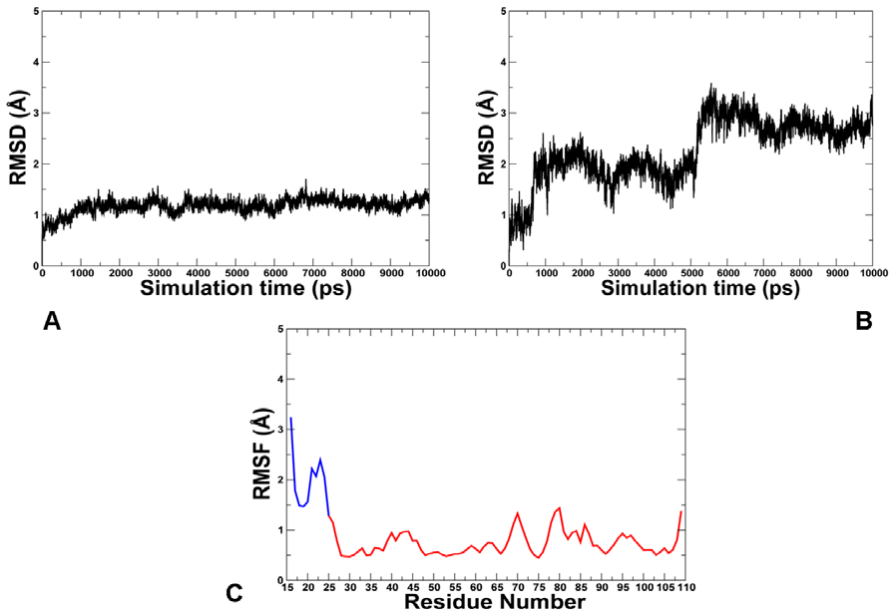
MD Simulations of the Phosphomimetic S17D MDM2 Form. MD simulations were carried out using a truncated lid form (residues 16–24). The RMSD fluctuations of the Cα atoms of the core MDM2 domain residues 25–109 (**A**) and the MDM2 lid residues 16–24 (**B**) obtained from 10 ns MD simulations of the phosphomimetic S17D form. (**C**) The RMSF values of the Cα atoms of the core MDM2 domain residues 25–109 (shown in red) and the MDM2 lid residues 16–24 (shown in blue) obtained from 10 ns MD simulations of the phosphomimetic S17D form.

**Figure 4:**
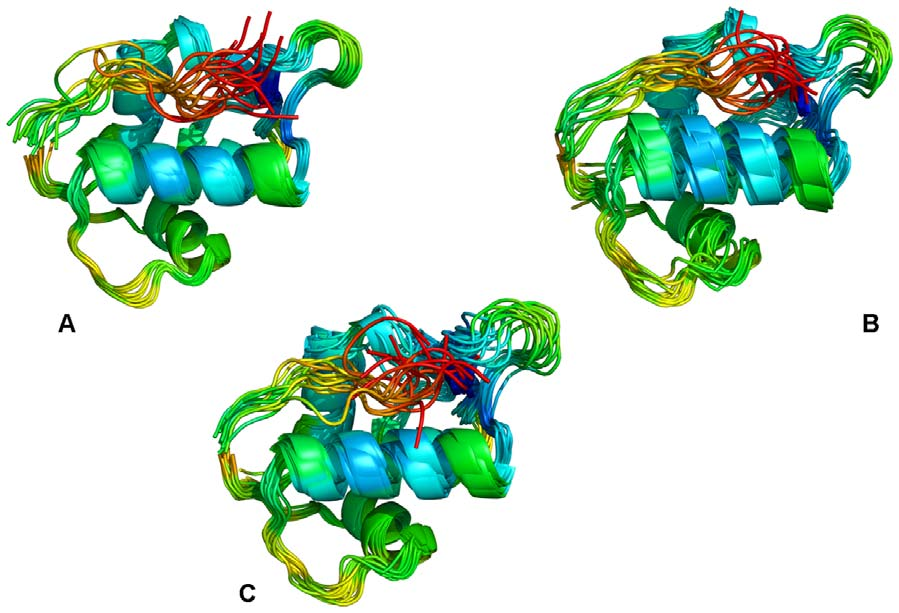
Mutation-induced Modulation of the MDM2 Conformational Ensembles: A Truncated Lid Model. Structural clustering of MD trajectories from simulations with a truncated lid (residues 16–24). The effect of phosphorylation and mutation-induced modulation of the conformational ensembles is illustrated for MDM2-WT (**A**), MDM2-pS17 (**B**) and MDM2-S17D (**C**). The apo-MDM2 (16–109) construct was used in all simulations. The representative MDM2 conformations from 10 dominant clusters were subjected to subsequent structural refinement by global minimization of the complete MDM2 structure. A ribbon-based representation of the MDM2 conformational ensembles was used. Coloring is according to the B-factors values (blue-to-red spectrum) reflecting protein nobilities of the MDM2 residues (from more rigid-blue regions to more flexible-red regions).

To quantify the effect of the lid size and better understand the mechanism of intramolecular MDM2 regulation, we first performed MD simulations using the truncated lid variant (residues 16–24) that was employed in a series of pioneering NMR studies [55], [57]. The time dependent evolution of MD trajectories was monitored using the root mean square deviation (RMSD) for all backbone atoms. Conformational mobility analysis revealed the enhanced stability of the MDM2 core domain (**Figure 2A**) and the lid (**Figure 2B**) in simulations of the phosphorylated MDM2-pS17 form. MD simulations were generally stable within a fluctuation range of RMSD  = 1Å for the core domain and RMSD  = 2Å for the lid. The protein flexibility variations were also evaluated from the root mean square fluctuation (RMSF) values of the backbone residues (**Figure 2C**). The thermal fluctuations of the MDM2 lid were reduced in the phosphorylated form and more evenly distributed among lid residues as evidenced by the RMSF values (**Figure 2C**). The observed restricted mobility of the lid segment 21-ASEQ-24 is in agreement with NMR studies [57], demonstrating that these residues may have order parameters as high as those of the MDM2 core domain. A different picture emerged from simulations of the phosphomimetic S17D form (**Figure 3**). Whereas the thermal fluctuations of the MDM2 domain remained stable within a similar fluctuation range of RMSD  = 1Å, the closed form of the S17D lid, that persisted during first 5 ns, transitioned towards the ensemble of more mobile, “semi-closed” conformations. (**Figure 3A, B**). Conformational mobility of these conformations was reflected in the relatively large RMSF  = 2.5 Å values of the mutated lid (**Figure 3C**). These simulations quantified the effect of posttranslational lid modifications and tested the initial conjecture, according to which phosphorylation of the S17 lid residue may enhance lid binding and thus inhibit the p53-MDM2 interactions [55]. On the simulation time scale, the equilibrium ensemble of apo-MDM2 with the truncated lid (residues 16–24) fluctuated primarily between semi-closed and closed states (**Figure 4A**), which agrees with the thermodynamic preferences towards the closed form [55], [57]. According to NMR-based study of (17−125) MDM2 [57], functional dynamics of the N-terminal lid can involve slow exchange (>10 ms time scale) between a dominant “closed” form and largely disordered “open” states. It is therefore consistent with the NMR experiments that on a nanosecond time scale of MD simulations the truncated lid would fluctuate near the closed state (**Figure 4**). We found that functional dynamics and the intramolecular interactions of the truncated lid could alter significantly in response to posttranslational modifications at S17. Indeed, MD simulations of the phosphorylated MDM2-pS17 form (**Figure 4B**) exhibited minor thermal fluctuations and structural stability of the pS17 lid, which reflected thermodynamic preferences towards a more ordered, “closed” lid form (**Figures 4B** **, S1**). On the other hand, the phosphomimetic S17D mutation may have a more subtle yet an appreciable functional effect on the lid dynamics, biasing the thermodynamic equilibrium towards an ensemble of more flexible, “semi-closed” conformations (**Figure 4C**). Mutation-induced changes in the equilibrium dynamics promote thermal fluctuations over a larger conformational space and are characterized by more frequently interconverting “closed” and “semi-closed” lid states (**Figures 4C** **, S2**). These observations are consistent with NMR experiments [55], [57] and demonstrate that the increased structural stability and the closed lid may be favored by the phosphorylated MDM2 form, whereas the phosphomimetic mutation may adjust thermodynamic preferences towards more structurally mobile conformations. Overall, our findings indicated that chemical modifications of the lid at phosphorylatable sites may serve as dynamic regulators of the thermodynamic equilibrium between preexisting conformational states of apo-MDM2.

**Figure 5:**
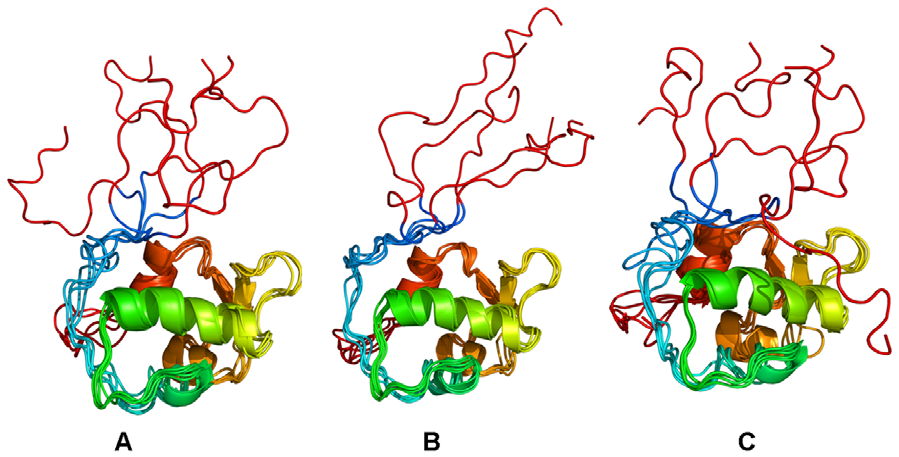
Mutation-induced Modulation of the MDM2 Conformational Ensembles: A Complete Lid Model. Structural clustering of MD trajectories from simulations with a complete lid (residues 1–24). The effect of phosphorylation and mutation-induced modulation of the conformational ensembles is illustrated for MDM2-WT (**A**), MDM2-pS17 (**B**) and MDM2-S17D (**C**). The complete apo-MDM2 (1–109) construct was used in simulations. The representative lid conformations are colored in red (residues 1–15) and cyan (residues 16–24). A ribbon-based representation of the MDM2 core domain was used.

MD simulations of the apo-MDM2 protein with the complete lid representation (residues. 1-MCNTNMSVPTDGAVTTSQIPASEQ-24) revealed a similar stability of the core domain (residues 25–109) (**Figure 5**). However, we observed very significant fluctuations of the lid conformations regardless of the chemical modifications at S17. These findings reflected the lack of meta-stable lid intermediates interacting with the MDM2 binding cleft. In agreement with the NMR studies [56], we found that a functional segment of the lid 21-ASEQ-24 may occasionally adopt helical-like conformations and form weak interactions with the binding cleft. However, these conformations were typically transient and short-lived, followed by a rapid dissociation of the lid from the binding site into a fully disordered state (**Figure 5**). Overall, the conformational lid ensemble of apo-MDM2 was highly diverse irrespective of the chemical state of the lid, where “open”, “partly open” and “partly closed” lid states displayed a considerable degree of disorder and interconverted on the simulation time-scale (**Figures 5**). In agreement with the latest biophysical investigations [63], our simulations of apo-MDM2 (1–125) indicated that the lid can form a mobile, “partly closed” form and participate in transient intramolecular interactions independent of the chemical transformations at S17. These results are also in accordance with the NMR studies [56], which suggested that the interactions between the N-terminal lid and the binding cleft of apo-MDM2 may be too short-lived to produce NOEs. The important computational investigation by Dastidar et al [90] has proposed that phosphorylation may drive the intramolecular interactions between the lid residue D11 and K94, thus stabilizing the closed lid form occluding the binding cleft. In our simulations, we could not detect a significant accumulation of meta-stable intermediate states, where the lid residues 2–18 formed interactions with the binding site. According to our findings, conformational ensembles were characterized by short-lived intramolecular interactions formed by residues 19-IPASEQ-24 largely irrespective of the chemical state of the lid at S17. At the same time the rest of the N-terminal lid was largely disordered and deviated away from the binding cleft (**Figure 5**). These results are supported by the NMR findings [56] which indicated the absence of long-range NOEs amongst residues 2–18 and ruled out the possibility for these lid residues to make stable interactions with the p53-binding groove. In addition, our findings may provide a plausible structural explanation why p53-MDM2 binding may be weakly dependent on the phosphorylation state as observed in biophysical studies with the complete apo-MDM2 construct [63]. Conformational ensembles of the truncated and complete lid exhibit considerable differences in the mobility and intramolecular interactions (**Figure S3A**). While the lid construct (16–24) could form a stable interface with the binding cleft and closely mimic p53-MDM2 interactions (**Figure S3B**), the full length lid (1–24) revealed a highly flexible ensemble of rapidly dissociating structures that typically form only weak, transient interactions in the binding site. Despite important differences in functional dynamics of apo-MDM2 as a function of lid length and composition (**Figure 5**), we found that the dynamic equilibrium between “closed”, and “semi-closed” (or “partly closed”) lid forms may be a fundamental characteristic of MDM2 regulatory interactions, which can be modulated by phosphorylation, phosphomimetic mutation as well as by the lid size. The mobile, yet partly closed lid form and weakened dependence on the phosphorylation can provide a rationale for binding of small p53-based peptides and inhibitors without direct competition with the lid dynamics. The central finding of our simulations revealed that such dynamic lid model is structurally and thermodynamically plausible, as well as consistent with diverse structural and biophysical experiments results [55]–[57], [63]. It is worth noting that our results do not support an alternative “open” model which suggested a positive regulatory role of the lid in p53-MDM2 interactions [61], [62].

### Hierarchy and Dynamics of Stabilizing Interactions: The Effect of Chemical Modifications in the MDM2 Lid

**Figure 6:**
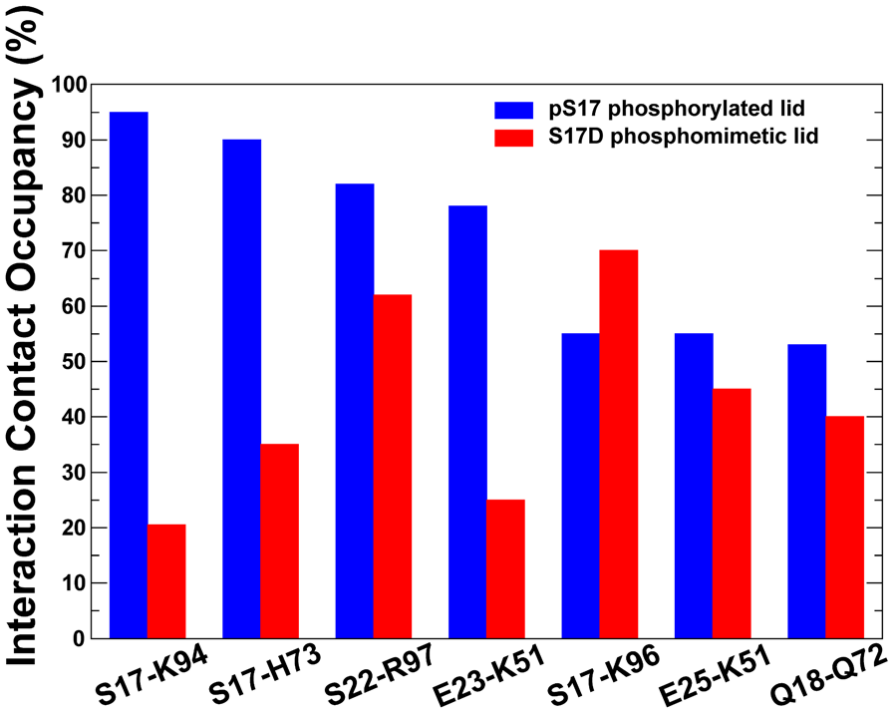
The Distribution and Occupancy of Specific Lid-Receptor Interactions. The distribution of the high occupancy contacts and salt bridges obtained from MD simulations of the phosphorylated MDM2-pS17 and phosphomimetic MDM2-S17D lid. The occupancies for pS17 are shown in blue filled bars and S17D in red filled bars. The analysis is focused on the dynamics of specific interactions formed by the lid with the core domain of MDM2. The intra-domain salt bridges are not included. The distance cutoff for hydrogen bonding is 3.50 Å and the angle cutoff is 120.00 degrees.

In the previous section, we concluded that the stability and dynamics of the intramolecular regulatory interactions may be better characterized and quantified using the truncated lid variant. Here, we analyzed functional dynamics of apo-MDM2 and the effect of chemical modifications in the lid to provide a detailed comparison and rationalization of NMR experiments where the respective apo-MDM2 variants (16–125) [55] and (17–125) [57] were studied. In general, lid residues 16-TSQIPASEQ-24 could form a number of favorable interactions in the MDM2 binding cleft, which is rich in charged residues (K51, K64, R65, Y67, K70, K94, R97, and K98). The high average occupancies of specific interactions formed by the lid with K94, H73 emerged as an important characteristic of the phosphorylated MDM2-pS17 form. These interactions are likely to be responsible for structural integrity and thermodynamic stability of the “closed” lid form (**Figure 6**). When S17 is phosphorylated, the negatively charged phosphate of pS17 can form strong salt bridges with K94. The stability of these interactions was also reflected in a rapid transition to the thermodynamically dominant “closed” form after 2 ns (**Figure S4A**). After a salt bridge between pS17 and K94 was established, the binding cleft of MDM2 was completely shielded by the lid and remained in this state throughout the simulation period. The hydrogen bond interactions formed by the pS17 phosphate group with K94, H73 and Q72 of the MDM2 core domain were also stable and maintained in the course of simulations (**Figure S4**). In particular, the most stable contacts were pS17-K94 (95% occupancy), pS17-H73 (90% occupancy), S22-R97 (82% occupancy) and E23-K51 (78% occupancy). Other specific contacts included S17-K96 (55% occupancy), E25-K51 (55% occupancy), and hydrogen bond between Q18 and the backbone carbonyl oxygen of Q72 (53% occupancy) (**Figure 6**). These interactions were further supported by the favorable contacts of the A21 backbone with the H96 and R97 residues. The hydrogen bonding network was accompanied by the favorable packing interactions formed by the lid with the L54, H96 and Y100 residues of the MDM2 binding cleft.

The pattern of stable specific interactions formed by the lid in the binding cleft was partly altered in simulations of the S17D mutant. Importantly, the occupancies of the key salt bridges S17D-K94, S17D-H73 and E23-K51, responsible for preserving the stability of the “closed” lid conformation, were markedly reduced (**Figure 6**). The weakened interactions with K94 and K51 were primarily due to the larger fluctuations of the S17D lid and the increased population of “semi-closed” conformational states. The occupancy of S17D-K94 was reduced to only 20% and E23-K51 to about 15%. The altered pattern of stabilizing interactions was also accompanied by repositioning of the hydrogen bonding formed by S17D towards the H96 side-chain (**Figure S4B**). The occupancy of the hydrogen bonding between Q18 of the lid and Q72 of the core domain was also reduced, thus reflecting the change in the interaction network and partial opening of the binding cleft. Additionally, the carbonyl oxygen of A21 was hydrogen bonded to H96 with 30% occupancy, and with the gate-keeper residue Y100 with 25% occupancy. As a result, the gate-keeper Y100 residue may become less constrained by the interactions with the MDM2 lid and adopt the “open” (“out”) state, thereby facilitating ligand access to the binding cleft [89]. These alterations in the network of stabilizing interactions reflected a shift in the thermodynamic equilibrium of MDM2-S17D towards the ensemble of “semi-closed” lid conformations. Collectively, these structural changes could promote a partial opening of the binding cleft making it accessible for inhibitors without interfering with the lid dynamics (**Figure 4C**).

We found that the dynamic equilibrium between “closed” and “semi-closed” lid forms is a common driver and determinant of MDM2 regulatory interactions. Thermal fluctuations between these forms were typically reflected in strengthening or weakening the key intramolecular interactions with K94/H73, without requiring global conformational changes. These results are in accordance with the NMR studies, suggesting that the phosphomimetic S17D can induce the “closed” lid form and block the MDM2 binding cleft from p53 [55], [57]. An alternative mechanistic model suggested that S17D could induce opening of the binding cleft by relocating the lid towards the interaction hot spot of solvent-exposed R97 and K98 residues [61], [62]. Computational studies [90] have also indicated that lid phosphorylation may induce ensembles of structurally distinct conformations driven by formation of salt bridges with K51, K94 or R97/K98 residues. Our results may explain conflicting experimental observations without assuming structural reorganization and migration of the lid away from the binding site. The proposed model suggested that large structural rearrangement of the lid may not be necessary to vacate the portion of the binding cleft sufficient for binding of small molecules. Structural reorganization of the lid required to establish hydrogen bonding of pS17 (or S17D) with a patch of the remote surface residues R97 and K98 may be disfavored on the thermodynamic grounds, irrespective of the lid size and composition. Indeed, conformational transformations to form a new intramolecular interface would inevitably involve breaking the binding site interactions and require desolvation of the charged and highly exposed surface residues.

### Functional Coupling of the S17 Phosphorylation and Y100 Gating in Modulating Conformational Ensembles of MDM2

It was previously proposed that gating dynamics of MDM2-Y100 between “in” and “out” conformations may control ligand access to the MDM2 binding cleft and influence the kinetics of p53 binding [89]. We conjectured that mutation-induced lid dynamics may be functionally coordinated with the gating dynamics of Y100 in modulating conformational ensembles and regulation of MDM2 binding. According to our results, the closed structure of the MDM2-pS17 lid could recruit the “in” conformation of MDM2-Y100 to optimize the interactions in the binding cleft (**Figure S5A**). In support of the functional coupling between the S17 and Y100 residues, we also found that mutation-induced bias of the S17D lid towards an ensemble of “semi-closed” conformations could be accompanied by rotation of the gate-keeper Y100 towards the open (“out”) conformation (**Figure S5B**). Hence, functional coupling between the lid dynamics and gating dynamics of Y100 can collectively coordinate opening of the binding cleft to allow access of p53-based peptides and small molecule inhibitors.

The MDM2 inhibitor nutlin-3 mimics the interactions of the p53 triad (L26, W23 and F19) in the binding site with all three hydrophobic pockets of the receptor [58], [59]. According to our results, the closed conformation of the phosphorylated pS17 lid can severely interfere with nutlin-3 binding (**Figure S6 A, B**). In particular, P20 of the closed lid would overlap with the bromophenyl inhibitor group occupying the p53-L26 binding pocket, and the nutlin-3 ethyl ether side chain, which is directed towards the p53–F19 pocket, would directly interfere with the interactions formed by pS17 and Q18 (**Figure S6B**). In contrast, the phosphomimetic S17D mutation may induce a partial opening of the binding cleft by moving away the stretch of lid residues 16–20 and altering the network of interactions formed by S17D and Q18 in the binding cleft (**Figure S6C**). In the closed lid conformation, the hydrophobic interactions with the first and second hydrophobic MDM2 pockets are mimicked by P20 and I19 of the phosphorylated lid. Upon S17D mutation, these lid residues tend to move away and vacate their positions in the binding site, thereby allowing the second bromophenyl group and the ethyl ether side chain of nutlin-3 to occupy their crystallographic positions (**Figure S6 C, D**).

The emergence of a “semi-closed” lid form in the equilibrium ensemble of MDM2-S17D can enable a partial opening of the binding cleft and allow for initial ligand entry. Additionally, the mobility of the lid conformations may provide means for subsequent optimization of MDM2 binding affinities with inhibitors. According to the proposed mechanistic scenario, binding of nutlins to MDM2 may proceed via search within the preexisting conformational ensemble of semi-closed MDM2 states for suitable low-energy receptor conformations. This may be followed by an induced-fit adjustment of the low-energy lid structures to optimize the intermolecular interactions. A model of “extended conformational selection” and conformational sampling of preexisting states [106],[107] may thus present a plausible mechanism of MDM2-mediated regulation by allowing proceeding through a hierarchy of conformational tiers that can be searched more efficiently than by random sampling of all possible conformations.

### Structural Analysis of the MDM2 Lid Interactions: Molecular Docking

To compare the equilibrium ensembles of apo-MDM2 with structural and energetic characterization of the intramolecular lid binding, we combined MD simulations and molecular docking. While equilibrium MD simulations may describe a mechanism of “conformational selection” between preexisting conformational states of MDM2, molecular docking and binding free energy analysis could more adequately model the induced-fit adjustment of the low-energy states. Consequently, the following specific objectives were addressed in docking experiments : (a) predict and validate the low-energy MDM2 structures by comparison with the equilibrium conformational ensembles; (b) characterize structural diversity of low-energy MDM2 structures and determine functional role of structural variations in the lid; (c) quantify pseudo-substrate interactions of the MDM2 lid and characterize the role of structural mimicry in regulation of MDM2 binding. Molecular docking of the lid (residues 16–24) with the core domain of the apo-MDM2 receptor (residues 25–109) was performed by using replica-exchange Monte Carlo simulations with the ensembles of multiple MDM2 crystal and NMR structures. To adequately simulate structure and energetics of the intramolecular lid binding in apo-MDM2, we employed a “partially fixed” lid model by constraining the MDM2 lid at a single Cα atom position of Gln-25. This model of a flexible lid considered a total of 37 torsional angles as independent variables during multiple docking runs. As a result, a very large conformational space was sampled in docking simulations to characterize the low-energy lid structures. It is worth noting that molecular docking of the complete lid (residues 1–24) presents a considerably more challenging problem that was attempted in our studies. However, the dramatic increase in the number of rotatable angles and the enormous size of the conformational space (>200 independent variables) combined with a highly flexible nature of the lid conformations make computational prediction of the low-energy states inherently uncertain and largely intractable. To quantify pseudo-substrate interactions of the MDM2 lid, we focused our analysis on structural and energetic predictions of the truncated lid binding as a function of posttranslational modification and mutation at S17.

**Figure 7:**
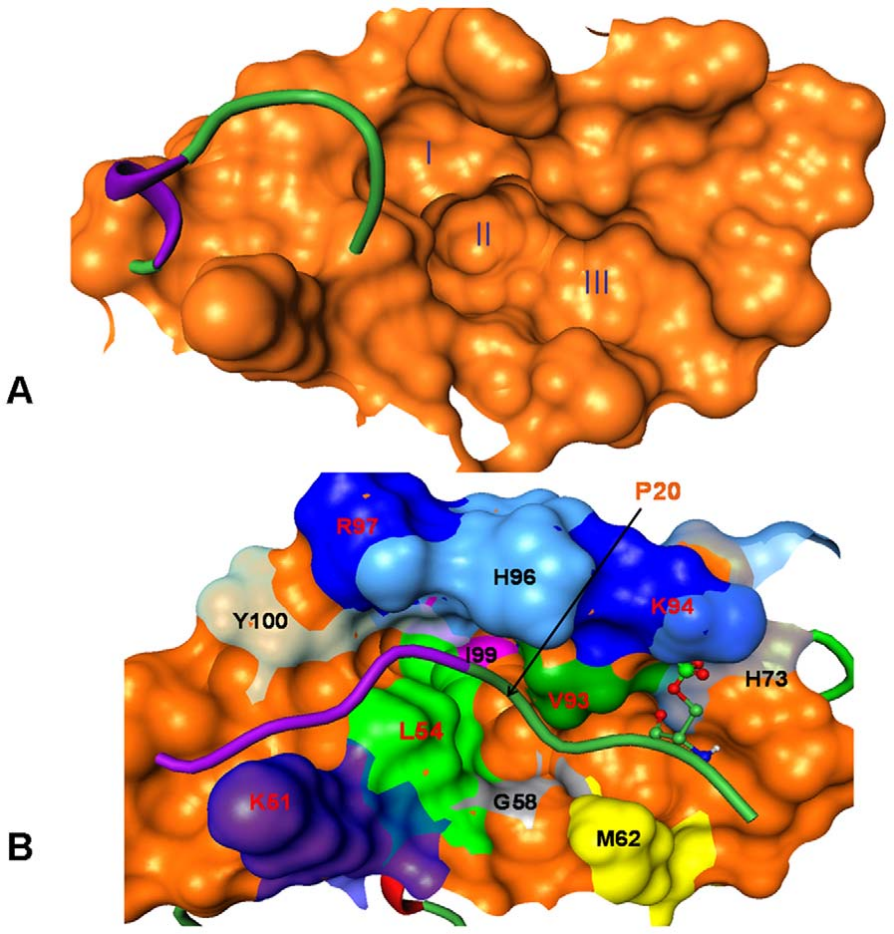
Folding-binding Coupling of the Phosphorylated pS17Lid. (**A**) The initial “open “conformational state of the lid shown over the p53 binding surface of MDM2 receptor. The three hydrophobic pockets (I, II, III) the receptor are occupied by respective p53 residues L26, W23 and F19 respectively in the p53-MDM2 complex. (**B**) The “closed” conformational state of the lid. Conformational changes in the MDM2 receptor and the phosphorylated lid upon binding are depicted. Structural restructuring and partial unfolding of the structured turn in the lid upon binding is highlighted in magenta. Positions of P20 and I19 on the MDM2 surface and phosphorylated S17 near K94 and H73 are also shown. Residues around the p53-binding site which undergo large conformational change upon lid binding are highlighted.

The initial unbound lid conformation before docking is shown in the binding site defined by the N-terminal amphipathic α-helix of p53 (**Figure 7A**). Chemical shift data of the MDM2 lid in the WT form have indicated that residues 21-ASEQ-24 could form a helical structure [57]. While simulations of the MDM2-WT supported these observations, we found that the phosphorylated lid may undergo a partial unfolding upon binding in the region 21-ASEQ-24 (**Figure 7B**). The strengthened interactions of the phosphorylated lid with all three pockets of the binding cleft and the increased ordering of residues 16–20 in the closed form may be fulfilled via partial unfolding of the helical structure for residues 21-ASEQ-24. The restricted movements of the pS17 lid, owing to the strong interactions with the receptor residues, may require the P20 residue (which is a strong helix breaker) to mimic interactions of p53-L26. This could restrict the range of allowed conformations and constrain the adjacent residues I19 and A21. Hence, our results suggested that folding-binding coupling in the fragment of the MDM2 lid may be modulated by the chemical state at S17, where the P20 residue may be strategically positioned as a key regulatory element controlling the helix structure. Hence, the increased order and stability of “closed” lid conformations is primarily determined by the strong binding preferences of the truncated lid.

**Figure 8:**
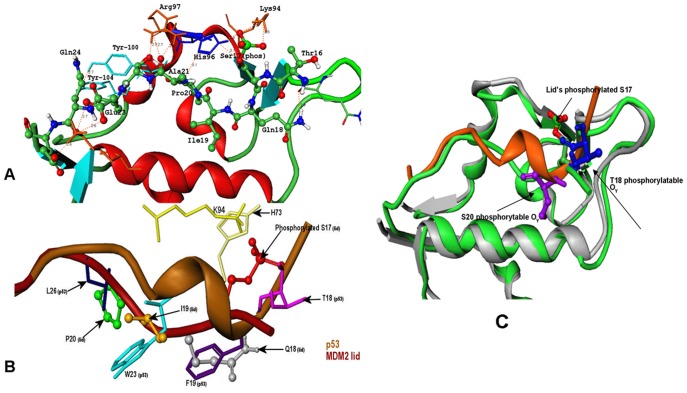
Structure-Functional Analysis of MDM2 and p53 Phosphorylated Sites. (**A**) A network of hydrogen bonding and hydrophobic interactions formed by the phosphorylated pS17 lid with the MDM2 core domain in the lowest energy structure. Functional importance of the conserved lid residues is illustrated. (**B**) An overlay of the p53 peptide (brown) with the phosphorylated flexible lid (dark red). The p53 residues L26 (in blue), W23 (in cyan), F19 (in magenta), and T18 (in pink) are shown in ‘sticks’, while corresponding residues of the phosphorylated lid when bound to MDM2 are shown in ‘ball-and-sticks’. Structural proximity of the phosphorylated pS17 of the lid to p53-T18 could be seen. (**C**) Structural proximity of the phosphate on the MDM2-pS17 lid (in ball-stick-model with atom-based coloring) and the phosphorylated positions on p53-T18 and p53-S20 (in ball-and-stick model colored in blue) is highlighted. The lowest energy structure of MDM2-pS17 (in green ribbons) is aligned with the crystal structure of the p53-MDM2 complex (in cyan ribbons).

**Figure 9:**
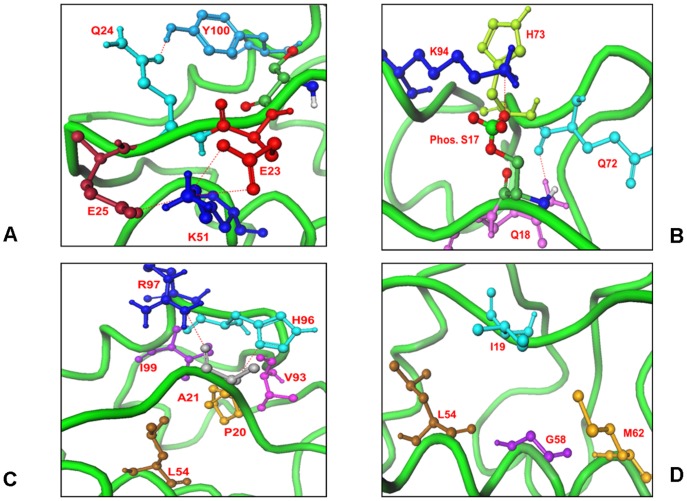
The Interaction Network of the Predicted Closed Form for the Phosphorylated pS17 Lid Interactions with MDM2. (**A**) The “anchoring” interactions of the phosphorylated lid formed by E23, Q24 and E25 residues K51 and Y100 core domain residues. E23 and E25 are shown in red sticks, K51 in blue sticks, Q24 and Y100 in light blue sticks. (**B**) The hydrogen bonds formed by pS17 and Q18 with K94, Q72 and H73 residues. PS17 is shown in ball-stick-model with atom-based coloring, K94 in blue sticks. (**C**) These interactions were further supported by the favorable contacts of P20 occupying the first hydrophobic pocket and A21 backbone with the H96 and R97 residues. The hydrogen bonding network is supported by the packing interactions of the lid with the L54, H96 and Y100 residues. (**D**) The MDM2 residues L54, L57, G58, I61, M62, V93, H96 and I99 are involved in the hydrophobic contacts of both p53 and pS17 lid. I19 is not projected towards the binding cleft; because of it restricted movement due to P20.

We characterized structural features of the low-energy phosphorylated lid structures using a comparative analysis of the two dominant lowest energy clusters (**Figure S7**). We found that the “closed” lid form was a common attribute shared by all major lowest energy clusters. Despite local differences in the bound lid conformations and receptor side-chains, the phosphorylated lid could strongly interact with the three hydrophobic pockets of the binding cleft (**Figure S7**). In agreement with MD simulations and NMR data, the specific interactions seen in all low energy clusters included hydrogen bonding of pS17 with K94, H73 and Q72 residues as well as the interactions formed by Q18 with the backbone carbonyl of Q72 (**Figures 8** **,** **9**). Consistent with MD simulations, pS17 lid conformations could preserve the key salt bridge formed by the phosphate group with K94 and specific interactions S22-R97 and E25-K51 that are critical for structural integrity of the closed lid form (**Tables 1** **and** **2**). The extensive network of hydrophobic contacts was also observed across all low energy clusters. A fundamental characteristic of the phosphorylated lid binding is a high degree of structural similarity between the low energy lid conformations and the p53 helix (**Figure 8**). In particular, MDM2-Q18 could mimic the hydrogen bond formed by p53-F19 and the pyrrolidine ring of MDM2-P20 could mimic the interactions of p53-L26 (**Figure 8B**). The interactions of the phosphorylated lid with K94, H73 and Q72 would bring the phosphate group of pS17 close to the position of p53-T18 (**Figure 8C**). According to these predictions and consistent with NMR studies [55], simultaneous phosphorylation of the truncated lid at S17 and p53 at T18 positions would bring the phosphate groups on these residues in a very close proximity, thereby likely interfering with the p53-MDM2 interactions. It was previously shown that phosphorylation of p53-T18 can weaken p53-MDM2 binding by ∼10-fold as compared to phosphorylation of p53-S20 [10], [11]. In agreement with these experimental findings, we observed that the phosphate position on pS17 in the low-energy structures and the position of p53-T18 in the crystal structure of the p53-MDM2 complex are in closer proximity to K94 and H73 than p53–S20 (**Figure 8C**). Hence, structural predictions suggest that a pseudo-substrate autoinhibitory mechanism may regulate p53-MDM2 binding. The key structural feature of the binding interface is the hydrogen bonding network formed by pS17 and Q18 with K94, Q72 and H73 residues (**Figure 9B**). In addition, structural position of P20 occupying the first hydrophobic pocket (**Figure 9C**) would likely make the carbonyl oxygen of the adjacent residue more electronegative, with the enhanced tendency for hydrogen bonding. Indeed, that carbonyl oxygen of A21 could form a strong hydrogen bond with R97 (**Figure 9C**). A partial constriction of the second binding pocket may also explain why I19 could not fully project inside the cleft (**Figure 9D**). A somewhat different pattern of the low-energy lid conformations emerged from docking simulations of the S17D mimetic lid (**Figure S8**). Unlike the phosphorylated lid, we observed that S17D may induce structural deviations of the residues 16–20 from their closed conformation. In one of the lowest energy states with a moderate conformational change (**Figure S8**), the interaction pattern was altered by breaking the hydrogen bonding with K94 and H73 and replacing it with the S17D-H96 interactions. However, other specific contacts between Q18 and Q72 were preserved, being largely responsible for the formation of the “semi-closed” lid form. A more drastic structural reorganization was observed in the alternative low energy cluster marked by the alteration of all three critical contacts with K94, H73, and Q72 (**Figure S8**). Some of these contacts may be only partly compensated by the contacts formed by S17D and Q18 with H96. In this low-energy structure, the phosphomimetic lid may open up a larger portion of the binding cleft by migrating most of the contacts away from the K94 and H73 residues toward H96.

**Table 1:** A comparison of the p53 and phosphorylated lid interactions with MDM2.

P53residue	MDM2 residue hydrogenbond contact	MDM2 residue hydrophobic contact	Lid residue	MDM2 residue hydrogenbond contact	MDM2 residue hydrophobic contact
E17	K94		pS17	K94,H73	
F19	Q72	I61,M62,Y67,V75	Q18	Q72	
L22		V93	I19		M62,V93
W23	L54	L57,G58,I61,V93	P20		L54,V93,I99
L26		L54,H96,V93	A21	H96,R97	
P27		M50,L54,Y100	S22	R97	
E28	K51		E23	K51	
N29	Y100,Y104		Q24	M50,Y100,Y104	
			E25	K51	

**Table 2:** Molecular mimicry of the p53-MDM2 interactions in the predicted structure of the phosphorylated pS17 lid.*

MDM2residue	MDM2atom	P53residue	P53atom	Lidresidue	Lidatom
H73	HD2	E17	OE2	pS17	O4
K94	2HZ	E17	OE1	pS17	O2
K94	2HZ	E17	OE2		
K94	1HZ			pS17	O4
Q72	O	F19	HD1	Q18	2HE2
L54	O	W23	HZ2		
K51	3HZ	E28	OE1	E23	OE2
K51	2HZ	E28	OE2	E23	OE1
K51	1HZ			E25	OE1
H96	ND1			A21	H
R97	2HH1	N29	OD1	S22	OG
R97	2HH2	N29	OD1	S22	OG
R97	2HH2			A21	O
Y100	HE12			Q24	OE1

* The table presents the residues and the corresponding atoms of MDM2 receptor which are involved in hydrogen bond formation with p53 residues and the phosphorylated lid residues.

**Figure 10:**
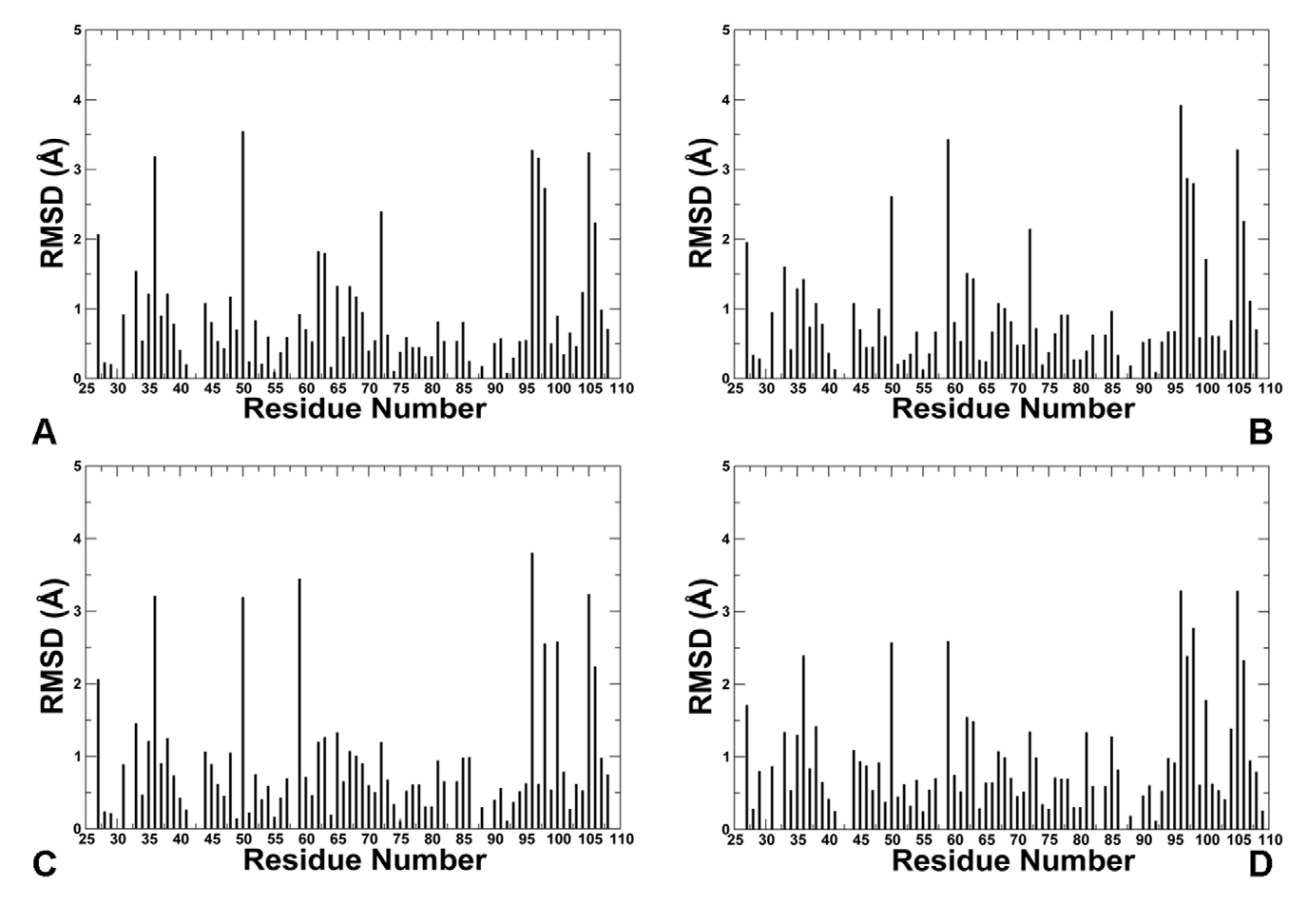
The Lid-based Modulation of Conformational Plasticity in the MDM2 Receptor: Analysis of Side-Chain Movements. Side chain movements in the MDM2 receptor upon binding of the phosphorylated pS17 lid in 3 lowest energy clusters from docking simulations are shown in (**A**)**,** (**B**) **and** (**C**)**.** (**D**) The RMSD values of the receptor side-chains averaged over 10 lowest energy clusters from docking simulations. The RMSD of the receptor side-chains were calculated using the crystal structure of the p53-MDM2 complex as the reference state.

The predicted structural models of the phosphorylated lid reflected the adjustment and refinement of the lid and core domain residues within the closed form. According to our results, the hydrophobic receptor residues near the binding pocket move side-chains to optimize their interactions with the phosphorylated lid (**Figures 10** **,** **11**). We observed the appreciable side chain movements (>2 Å) in M50, K51, M62, Q72, K94, H96, R97, K98 and Y100 residues that collectively adjusted their positions to optimize interactions with the closed pS17 lid. Structural plasticity of the binding site residues may be determined by coordinated movements involving relatively small changes in the protein backbone and large variations of the side–chains (**Figures 10** **,** **11**). According to our results, this may be sufficient to optimally accommodate structural and chemical variations in the lid. Structural similarity between the receptor backbone in the bound lid structures and the p53-MDM2 complex is in agreement with NMR studies [57]. Hence, our results are consistent with the notion that global conformational changes in the MDM2 receptor may not be required for binding of p53-derived peptides and small molecule inhibitors [57], [63].

**Figure 11:**
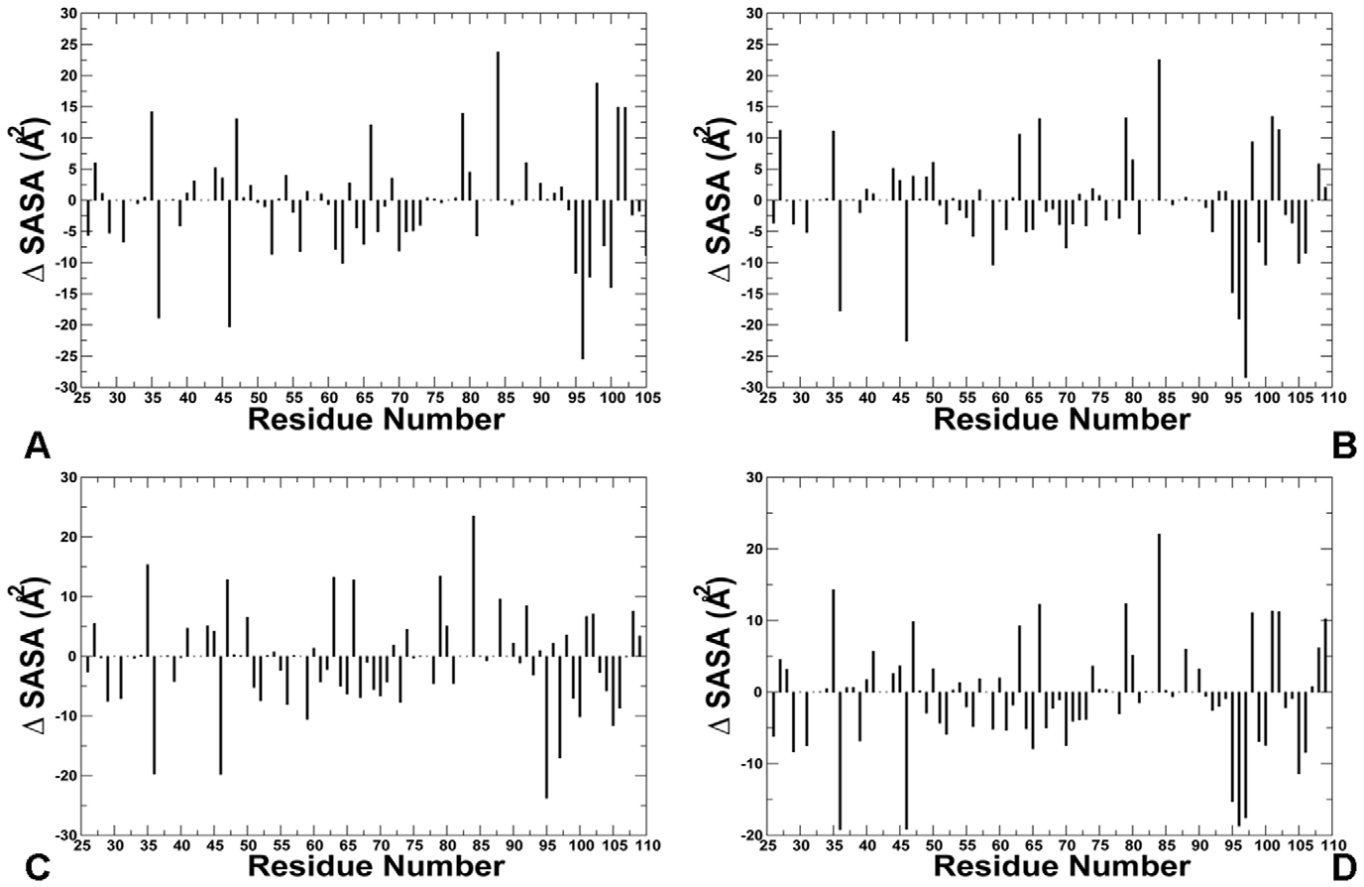
The Lid-based Modulation of Conformational Plasticity in the MDM2 Receptor: Analysis of Solvent Accessible Surface Area Changes. Change in the percentage of the accessible surface area (ASA) of the receptor residues upon binding of the phosphorylated lid in 3 lowest energy clusters from docking simulations (**A**), (**B**) and (**C**). (**D**) The change in the percentage of ASA for the receptor side-chains averaged over 10 lowest energy clusters from docking simulations. The crystal structure of the p53-MDM2 complex was used as the reference structure. Positive (negative) values indicate that the residues get more exposed (buried) upon binding of the lid as compared to their position in the crystal structure of the p53-MDM2 complex.

### Molecular Mimicry of Pseudo-Substrate Lid Interactions

In this section, we characterize the interaction networks and key lid residues involved in the intramolecular regulation of MDM2. This analysis allowed to rationalize at the atomic level the observations from NMR experiments where the respective apo-MDM2 variants (16–125) [55] and (17–125) [57] were investigated. Structural similarity between the p53-MDM2 interaction network and the phosphorylated lid interactions in the binding cleft (**Tables 1** **,** **2**) is a key functional feature reflecting conservation of the interaction networks with the MDM2 receptor (**Tables 1** **,** **2**). The electrostatic and hydrogen bonding interactions made by E17, F19, E28 and N29 of p53 are replaced respectively by pS17, Q18, E23 and Q24 residues of the lid. The hydrophobic p53 interactions in the first and second hydrophobic pockets are mimicked P20 and I19 of the phosphorylated lid. Many common MDM2 residues, including L54, L57, G58, I61, M62, V93, H96 and I99, are involved in the hydrophobic contacts with p53 and the pS17 lid. Moreover, the “anchoring” interactions that position the phosphorylated lid in the binding site to mimic p53-helix are also preserved. These interactions that are formed by Q24 with M50 and E25 with K51 may anchor the rest of the lid and provide an additional stabilization of the closed lid structure (**Tables 1** **,** **2**).

**Figure 12:**
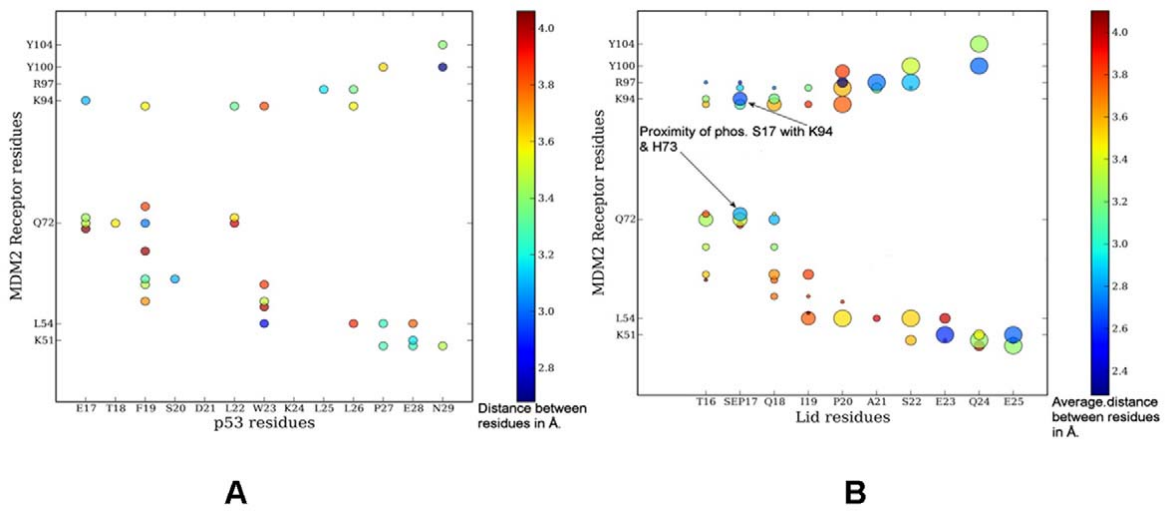
The Contact Maps of the p53-MDM2 and MDM2-pS17 Interactions. (**A**) The contact map of the p53 residues with the HDM2 receptor in the crystal structure of the p53-MDM2 complex (PDB ID 1YCR). (**B**) The contact map of the truncated pS17 lid with the MDM2 receptor in 5 lowest energy structures models. Residues are considered in contact when the minimum inter-residue distance of all pairs of heavy atoms is <4.2Å. A comparison indicates a similarity in the nature of the contacts and the contact distances. Larger circles indicate a more probable interaction. The color coding of the circles indicate the minimum inter-residue distance between the residues and the size signifies the number of conformation forming the same interaction. Plot has been generated using R package.

We also compared the contact map of the p53-MDM2 interactions in the crystal structure with the contact map of the phosphorylated lid interactions in 5 different low energy conformational clusters (**Figure 12**). These contact maps revealed a striking similarity in the interaction networks formed by p53 and the phosphorylated lid conformations with the MDM2 receptor. The circle size signifies the number of structures forming exactly the same interaction, and it could be observed that the most consistent contacts made by the phosphorylated pS17 are with H73, K94, Q72 and only in one low-energy solution pS17 is in the contact with H96 (**Figure 12**). In contrast, the largest variation could be seen in the binding pattern of the N-terminal residue T16. According to the p53-MDM2 interactions network topology, p53-T18 is in the close proximity of Q72, while S20 is in the contact with M62.

A comparison of the contact maps could also illustrate that the phosphorylation of Ser17 in the truncated lid would have a more pronounced effect on p53-MDM2 binding when p53-T18 is phosphorylated as compared to the phosphorylation effect at p53-S20. The contact maps also indicated that similar interactions can be formed with the same set of evolutionary conserved MDM2 residues [81], [82]. These results supported the long-standing notion that molecular mimicry of p53-MDM2 interactions [81], [82] is an important mechanism driving binding of high-affinity mimetics and small molecule inhibitors [96]–[105]. Our results indicated that the intramolecular binding of the functional lid variant (residues 16–24) may utilize elements of molecular mimicry to act as a natural antagonist to p53 binding. It is worth noting that the representative conformational ensemble obtained in simulations with the complete lid construct displayed a shortage of structural similarity with the p53-MDM2 interactions (**Figure S3B**). This observation is consistent with biophysical studies of the complete apo-MDM2 construct (1–109) [63] and may explain why this lid form can be readily displaced by p53-based peptides irrespective of the phosphorylation state.

### Energetic Basis of the MDM2 Lid Modulation: Binding Free Energy Analysis

The first principles analysis of MDM2 binding involves a comparative characterization of p53 binding thermodynamics with a panel consisting of the complete MDM2 receptor (residues 1–109), truncated MDM2 protein (residues 16–109), and the MDM2 core domain (residues 25–109) as well as phosphorylated and mutated MDM2 analogues. However, a full implementation of such approach is computationally intractable for various reasons, including (a) the lack of crystal structures of the p53-MDM2 complex with the lid present (in either truncated or complete form); and (b) the inherent uncertainty in computing peptide binding free energies in the presence of long and highly variable lid construct. To understand structure and energetics of the intramolecular lid-MDM2 interactions, we modeled the apo-MDM2 structure (residues 16–109) as a hypothetical ligand-protein complex, where the truncated lid (residues 16–24) was considered as a partially constrained ligand (at the Cα position of Gln-25) and the MDM2 core domain (residues 25–109) was used as the protein. We computed binding free energies of the lid as a function of phosphorylation state and compared these estimates with binding free energies of the p53-MDM2 complex.

A simplified knowledge-based model and a detailed MM-GBSA model were employed (**Tables 3** **,** **4**). Binding free energies estimates using the empirical scoring model averaged the energy contributions over the low energy clusters. ΔG_binding_ of the p53-MDM2 complex was −5.72 kcal/mol using the knowledge-based scoring function (**Table 3**) and −7.3 kcal/mol using the MM-GBSA energy function (**Table 4**) which is in agreement with the experimental data (ΔG_binding_ −6.6 to −8.8 kcal/mol) [82]. The estimated binding free energy of the pS17 lid with the MDM2 core domain (ΔG_binding_ ≈−10.0 kcal/mol) suggested that the phosphorylation at S17 would give rise to the energetically more favorable closed form of the truncated lid that may displace p53 binding on thermodynamic grounds (**Tables 3** **,** **4**). The energetic decomposition of the binding free energy differences revealed that the free energy factors may act concertedly to enhance the binding affinity of the phosphorylated lid. The binding of the p53 helix is mainly driven by the van der Waals interactions formed by F19, W23 and L26 of p53. Although the two hydrophobic residues I19 and P20 of the phosphorylated lid mimic the p53-MDM2 interactions, the hydrophobic contribution of p53 binding is more favorable, partly due to insufficient penetration of I19 into the binding cleft. Despite the ordered conformation of the phosphorylated lid, the entropic penalty was found to be comparable to p53 using the knowledge-based scoring function (**Table 3**) and somewhat larger according to more accurate MM-GBSA estimates (**Table 4**). These results indicated that the truncated lid can adopt optimal structural arrangements in the binding cleft without a large entropic penalty. The hydrogen bonding, the electrostatic and the van der Walls interactions were more favorable for the pS17 lid, suggesting that specific lid interactions in the binding cleft may be a critical energetic factor in allowing to displace p53-MDM2 binding. These results agreed with the previous experimental studies [76], [77] and computational investigations [82], [95], suggesting an important role of the van der Waals contribution in driving binding affinities with MDM2.

**Table 3:** Binding free energy calculations of p53-MDM2, pS217-MDM2 and S17D-MDM2 using a knowledge-based energy function.*

Binding Energy	p53-MDM2	MDM2-pS17	MDM2-S17D
ΔG_hydrophobic_	−7.02	−5.85	−4.56
ΔG_hydrogen bond_	−0.02	−9.64	−5.32
ΔG_vdW_	−36.94	−36.51	−30.12
ΔG_electrostatic_	−1.72	−0.44	−0.87
ΔG_desolvation_	6.81	8.57	5.79
ΔG_entropy_	33.17	33.1	28.53
ΔG_total_	−5.72	−9.96	−6.55

* All energies are in kcal mol^−1^. The contributions of the free energies are defined in the Materials and Methods section.

**Table 4:** MM-GBSA calculations of binding free energies for p53-MDM2, pS217-MDM2 and MDM2-S17D.*

Binding Energy	p53-MDM2	MDM2-pS17	MDM2-S17D
ΔE_ele_	−467.3	−487.4	−470.9
ΔE_vdw_	−83.8	−88.2	−75.9
ΔE_int_	8.6	5.3	6.7
ΔE_MM-gas_	−542.5	−570.3	−540.1
ΔG_gb-nb_	−16.4	−17.5	−18.4
ΔG_gb-pol_	484.5	504.6	488.4
ΔG_gb-total_	468.1	487.1	470.0
TΔS_total_	67.1	70.6	62.3
ΔG_total_	−7.3	−12.6	−7.8

* All energies are in kcal mol^−1^. The contributions of the free energies are defined in the Materials and Methods section.

We found that the electrostatic and hydrogen bonding terms favoring the lid closure over the binding cleft could not be completely screened by the unfavorable desolvation contribution (**Tables 3** **,** **4**). The binding preferences of the pS17 lid are determined by a “concerted effort” of both hydrogen bonding and the van der Waals interactions. By adopting a thermodynamically favorable closed form, the truncated lid may act as a small ligand and effectively compete with p53 (17–29) binding because of comparable entropic penalty upon sequestration from MDM2. The binding free energy of the phosphomimetic S17D lid was also evaluated using the empirical scoring based on the low-energy clusters of docked conformations. ΔG_binding_ was in the range between −6.55 kcal/mol, when using the empirical scoring function (**Table 3**), and −7.8 kcal/mol, when using the MM-GBSA model (**Table 4**). The reduced affinity of S17D as compared to the phosphorylated pS17 analog was mainly due to the weakening of the hydrogen bonding, the electrostatic and the van der Waals interactions with the binding cleft (**Tables 3** **,** **4**). These estimates reflected the increased structural mobility of the low-energy lid conformations and a partial loss of the interactions with the binding site. According to our results, this loss could not be offset by the reduced entropy penalty and the lowered desolvation cost upon binding. Hence, while the enthalpy component may drive favorable binding of pS17, the perturbed balance of the enthalpy and entropy contributions could lower the affinity of the phosphomimetic lid, and make it comparable to the a binding affinity of the p53-MDM2 complex. Our results confirmed the universality of the enthalpy-entropy compensation principle that may explain binding preferences of MDM2 for the phosphorylated lid over p53. The observed pattern of changes in the enthalpy-entropy compensation upon chemical modifications in the lid is reminiscent of similar findings made from isothermal titration calorimetric studies [98], where more favorable entropy of binding for constrained peptidomimetics was largely offset by a reduced enthalpy contribution. Conversely, the entropy loss, often seen in binding of linear p53-derived peptides, may be compensated by optimized intermolecular contacts and enhanced enthalpy contribution. Similarly, the high mobility and weakened intramolecular interactions seen in simulations with the complete lid should favor its displacement by p53-based peptides of different length as evidenced from latest biophysical studies [63].

## Discussion

In this study, we determined that the dynamic equilibrium between “closed”, and “semi-closed” lid forms may be an important characteristic of MDM2 regulatory interactions, which can be modulated by phosphorylation, phosphomimetic mutation as well as by the lid size. Our results revealed that these factors may regulate p53-MDM2 binding by fine-tuning the thermodynamic equilibrium between preexisting conformational states of apo-MDM2. In agreement with NMR studies, the effect of phosphorylation is more pronounced with the truncated lid variant, favoring the closed form of the MDM2 lid. The dominant “semi-closed” lid form and weakened dependence on the phosphorylation observed in simulations with the complete lid can provide a rationale for binding of small p53-based peptides and inhibitors without direct competition with the lid dynamics.

We have shown that “extended conformational selection model” is a robust indicator of MDM2 functional dynamics and may adequately describe mechanism of MDM2 regulatory interactions. According to the proposed model, molecular mechanisms of MDM2 binding could be regulated via mutation-induced lid modulation of preexisting conformational states [106], [107]. Our results supported the notion that the organization of the MDM2 conformational landscape may be separated in different tiers of protein fluctuations in response to chemical modifications and ligand binding. As a result, conformational changes in MDM2 may be hierarchic and combine “conformational selection” among preexisting conformational ensembles, with a subsequent sub search and induced-fit adjustment. Based on these findings, we propose that the “extended conformational selection” model [106], [107] of the lid dynamics may provide a plausible and unifying platform for rationalization of the existing data. From a thermodynamic perspective, the conformational selection model and induced fit can be considered as complementary models, as due to thermal fluctuations there is always some degree of the induced fit on the atomic scale of molecular interactions. In the framework of this model, the dynamics of flexible segments may be often separated from the rest of the protein and evolve on a different time scale. These functional regions may include pseudo-substrate lid motifs that operate in a range of allosterically regulated enzymes. According to the fundamental assertion of the extended conformational selection model, these fragments (termed ‘discrete breathers’) may regulate a population-shift between conformational ensembles that accompany binding processes through changes in their mutational status [106]. Our results also supported the notion that mechanisms of molecular mimicry and conformational selection may have been adopted by MDM2 as organizing principles to utilize structural plasticity for binding to a wide range of protein clients. These conclusions are consistent with the fact that global conformational changes in the MDM2 receptor may not be required for functional diversity of high affinity binding molecules, which may greatly facilitate the potential of MDM2 to serve as a hub in protein networks and accommodate interacting partners.

We have shown that an “extended conformational selection” model may explain structural and enzymological data based on computational analysis of the N-terminal MDM2 domain, thus bypassing for now the challenge of modeling the full length MDM2 [90]. However, conformational flexibility of MDM2 and the existence of multiple binding domains have highlighted the important role of allostery in regulating the complexity of intra- and inter-domain communication. Structurally discrete but interdependent functional domains of MDM2 are allosterically linked through structural and dynamic changes that are currently unknown due to lack of structural information. It is tempting to suggest that the “extended conformational selection model” may be potentially fruitful to explain functional dynamics of the interdomain MDM2 regulation. These findings corroborate with the recent results pointing to a common role of conformational selection principles in a variety of biological systems [108]–[111]. It is possible that a range of allosterically regulated protein hubs involved in binding with diverse partners, including MDM2 receptors, can adapt to protein clients and ligands by using a mechanism of equilibrium switching [106], [107]. The results of this work may be useful for probing functions and modeling mechanisms of MDM2 regulation in signaling cascades modulated by posttranslational modifications on the p53 regulators. Further studies connecting diverse experiments with computational studies of allosteric signaling and network biology would likely have broad implications in the development of novel anti-cancer therapies.

## Materials and Methods

### MD Simulations

We have performed a total of 6 independent 10 ns MD simulations of the N-terminal domain of MDM2. These simulations were carried out separately using a complete structure of the lid (residues 1–24) and truncated functional lid (residues 16–24) for the MDM2-WT, the phosphorylated form MDM2-pS17, and the phosphomimetic form MDM2-S17D. The initial conformations of the lid conformations were obtained from the NMR solution ensemble of apo-MDM2 (PDB ID 1Z1M) [56]. This ensemble includes a diverse range of states, including both open and closed lid conformations [56]. To avoid a potential bias in initial conditions during MD simulations, we selected MDM2 structures with a disordered lid conformation (corresponding to states 4, 8,9,10 and 11 according to their number in the NMR ensemble [56]). The missing and unresolved residues were modeled using the program MODELLER [112], [113]. The N-terminal lid variant (16-TSQIPASEQ-24) was capped at the terminus by the acetyl cap (CH3-CO-). MD simulations were carried out using NAMD 2.6 [114] with the CHARMM27 force field [115], [116] and the explicit TIP3P water model as implemented in NAMD 2.6 [117]. The VMD program was used for the preparation and analysis of simulations [118], [119]. The employed MD protocol was described in full details in our earlier studies [120]–[122]. MDM2 structures were solvated in a water box with the buffering distance of 10 Å. Assuming normal charge states of ionizable groups at pH 7; counter-ions were added to neutralize the total charge of the system.

The system was subjected to initial minimization for 20,000 steps (40ps) keeping protein backbone fixed which was followed by 20,000 steps (40ps) of minimization without any constraints. Equilibration was done by gradually increasing the temperature in steps of 20 K starting from 10 K until 310 K. At each step 15000 steps (30 ps) equilibration was run keeping a restraint of 10 kcal·mol^−1^·Å^−2^ on protein alpha carbons (C_α_). Thereafter the system was equilibrated for 150,000 steps (300ps) at 310K (NVT) and then for further 150,000 steps (300ps) at 310 K using Langevin piston (NPT) to achieve uniform pressure. Finally the restrains were removed and the system was equilibrated for 500,000 steps (1ns) to prepare the system for simulation. An NPT simulation was run on the equilibrated structure for 10 ns keeping the temperature at 310 K and pressure at 1 bar using Langevin piston coupling algorithm. The integration time step of the simulations was set to 2.0 fs, the SHAKE algorithm was used to constrain the lengths of all chemical bonds involving hydrogen atoms at their equilibrium values and the water geometry was restrained rigid by using the SETTLE algorithm. The van der Waals interactions were treated by using a switching function at 10Å and reaching zero at a distance of 12Å. Non bonded lists were updated after 25 steps. The particle-mesh Ewald algorithm (PME) implementation in the NAMD program was used to simulate long range electrostatic interactions. Then production runs were performed for 10 ns (each trajectory) with trajectory frames saved at 2ps interval. The Pymol program was used for visualization of protein structures (The PyMOL Molecular Graphics System, Version 1.2r3pre, Schrödinger, and LLC).

### Replica-exchange Monte Carlo Docking Simulations

The Protein Data Bank (PDB) [123] contains a number of crystal structures of the N-terminus domain of MDM2 (or its homologues) in the bound and apo forms. However, the N-terminal lid conformation (residues 1–24) is not resolved in most of these structures. To study the effect of lid binding on the p53-MDM2 interactions, we have used the co-crystal structure of the p53-MDM2 complex (PDB ID 1YCR) [45] as the initial conformation of the N-terminal MDM2 domain, and attached the lid residues from the crystal structure of MDM2 with the benzodiazepine inhibitor (PDB ID 1T4E) [76] to the N-terminus after structural alignment of the two structures. All publically available crystal structures of the MDM2 protein from PDB were used to categorize the conformational space of MDM2, including the crystal structure of xenopus MDM2 bound to the transactivation domain of p53(PDB ID 1YCQ) [45]; the crystal structure of human MDM2 bound to the transactivation domain of p53(PDB ID 1YCR) [45]; the crystal structure of human MDM2 with an imidazole inhibitor nutlin (PDB ID 1RV1) [58]; the crystal structure of human MDM2 in complex with a beta-hairpin PDB ID 2AXI) [64]; the crystal structure of the MDM2 complex with an 8-mer p53 peptide analogue (PDB ID 2GV2) [65]; the crystal structures of human MDM2 in the complex with a p53-derived peptide (PDB ID 1T4F) and a benzodiazepine inhibitor (PDB ID 1T4E) [76]; an ensemble of 24 NMR structures of apo-MDM2 (PDB ID 1Z1M) [56]; and the NMR structure of a complex between MDM2 and a small molecule inhibitor (PDB ID 1TTV) [60]. We have also expanded a spectrum of low–energy conformational states which may mimic a range of protein equilibrium fluctuations near the native structures. The MDM2 structures were initially prepared by adding the hydrogen atoms and optimizing their positions. Side chain ionization states were adjusted to pH 7.0. The computational procedure uses the penultimate rotamer library [124] to build and optimize multiple rotamers for the critical MDM2 binding site residues, including L54, Leu57, I61, M62, Y67, Q72, V75, F86, F91, V93, I99, Y100, and I103. The energies of the modified protein are optimized using a simple self-consistent procedure in which optimization of the rotamer position for a modified residue is followed by the same procedure for the next mutated residue until convergence is achieved [125]. We modeled the apo-MDM2 structure (residues 16–109) as a ligand-protein complex, where the truncated lid (residues 16–24) was used as a partially constrained ligand (at the Cα position of Gln-25) and MDM2 (residues 25–109) was used as the protein. The N-terminal lid variant (16-TSQIPASEQ-24) was capped at the terminus by the acetyl cap (CH3-CO-).

Molecular docking simulations were performed using replica-exchange Monte Carlo simulations with the ensembles of multiple MDM2 crystal structures. To expand the conformational ensemble of apo-MDM2 and generate low–energy conformational states near the native structure, we employed a self-consistent rotamer optimization [125]. The details of the docking protocol were detailed in our previous studies [126], [127]. In brief, the molecular recognition energetic model used in dynamics simulations includes intramolecular energy terms, given by torsional and nonbonded contributions of the DREIDING force field [128], and the intermolecular energy contributions calculated using the AMBER force field [129] combined with an implicit solvation model [130].The dispersion-repulsion and electrostatic terms were modified to include a soft core component that was originally developed in free energy simulations to remove the singularity in the potentials and improve numerical stability of the simulations [131]. Replica-exchange simulations used 1,000 replicas of the system (corresponding to 1,000 different snapshots) attributed respectively to 1,000 different temperature levels that were uniformly distributed in the range between 5300 K and 300 K. The crystal structures, the ensembles of near-native crystal structure conformations and MD simulations samples were replicated and uniformly distributed as a total of 1,000 replicas to 1,000 different temperature levels. Monte Carlo moves were performed simultaneously and independently for each replica at the corresponding temperature level. A process of swapping configurations was repeated 100 times after each simulation cycle for all replicas. For the optimal performance a number of free parameters such the appropriate temperature distribution, range of temperatures, number of Monte Carlo sweeps at each temperature and number of swaps between different temperature levels after each cycle were optimized as previously documented [126], [127]. The lid conformations and orientations were sampled in a parallelepiped that encompasses the crystal structures of the MDM2 complexes with a 10.0 Å cushion added to every side of the box surrounding the binding interface. The MDM2 core domain structures were held fixed in their minimized crystallographic conformations, while the rigid body degrees of freedom and the rotatable angles of the lid were treated as independent variables during docking.

### Binding Free Energy Analysis

The binding free energy of each protein-ligand complex was estimated using the knowledge-based scoring function [132] which is a linear combination of the hydrophobic energy contribution, the electrostatic energy component, desolvation energy of polar and nonpolar groups, the van der Waals energy contribution, the internal energy term and the entropy contribution corresponding to the loss of translational and rotational degrees of freedom. The solvation terms were fitted to experimental solvation free energies and the entropy terms were derived from sublimation thermodynamic data. The low energy lid conformations and MDM2 receptor conformations were minimized together as a single complex by a truncated Newton optimization algorithm and a gradient cutoff of 0.1 kcal/mol as the termination criteria. Binding free energy calculations were also carried out using the molecular mechanics (MM) AMBER force field [133] and the solvation energy term based on continuum generalized Born and solvent accessible surface area (GB/SA) solvation model [134]–. In MM-GBSA calculations, we employed AMBER99 force field. The total free energy was given as
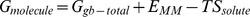


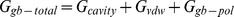



In the GB/SA model, the 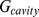 and 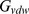 contributions are combined together via evaluating solvent-accessible surface areas:
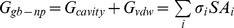
where 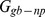 is the nonpolar solvation term derived from the solvent-accessible surface area (SA). 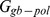 is the polar solvation energy which is computed using the GB/SA solvation model. A residue–based cutoff of 8Å is set for computing nonbonded van der Waals interactions and 20 Å residue–based cutoff is used for computing electrostatic interactions the values of the interior dielectric constant and the exterior dielectric constant were set to 2 and 80, respectively. The surface tension coefficient was set to 0.0072 kcal·mol^−1^·Å^−2^. The solute entropy is the sum of the translational, rotational and vibrational entropy contributions.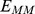 is the molecular mechanical energy of the molecule and includes the electrostatic interactions 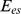, the van der Waals contributions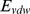, and the internal strain energy 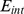. The contributions of the ligand–protein interaction energy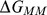, strain energy 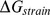 and solvation energy 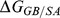 to the total binding free energy can be then determined as follows.



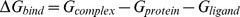


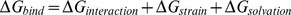



The entropic contribution was calculated within the AMBER module NMODE, where the vibrational entropy contribution TSvib was evaluated from classical statistical mechanics formula. Each snapshot was minimized for 100,000 steps in the presence of a distance-dependent dielectric of 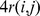 (where 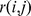 is the distance between two atoms) until the root-mean-square of the elements of the gradient vector was less than 5×10−^5^ kcal mol^−1^ Å^−1^. 100 representative snapshots were used to estimate the entropy contribution. The binding free energy of complexes was calculated as follows:
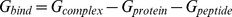



A practical implementation of this approximation involved evaluation of the free energies for 1,000 snapshots selected at 10 ps interval along MD trajectories. The total binding free energy values were obtained by averaging calculated contributions over 1,000 simulation snapshots at T = 300 K**.** The binding free energy evaluations could be performed using either separate trajectories of the MDM2 complexes, MDM2 core domain and the lid or a single trajectory [138]. We have used a single trajectory protocol where the structures for the unbound, lid-free MDM2 core domain and the lid structures were obtained by separating the MDM2 and lid coordinates, followed by an additional minimization of the unbound protein and lid.

### Structural Similarity Analysis

We have also employed structural clustering and similarity analysis of the lid conformations generated in simulations. Structural similarity metric is based on spatial proximity of the lid atoms and the atom type. We distinguish hydrogen bond donors, hydrogen bond acceptors, hydrogen bond donors and acceptors and nonpolar atoms. The atom type compatibility 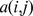 is assigned a value between 0.0 and 1.0, with the compatibility between two atoms of the same type defined to be 1.0 that between donor and acceptor atom is 0.0. The spatial proximity between two atoms 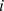 and 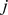 is evaluated with a Gaussian function 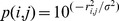 and 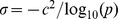 where 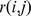 is the distance between atoms 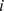 and 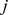; 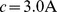
and 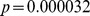 are the cutoff distance and proximity threshold respectively. We calculate a descriptor 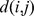 from the spatial proximity and the atom type compatibility:



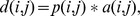


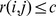




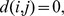


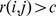


An atom descriptor 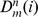 for atom 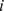 in molecule 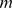 is then calculated by summation over all 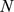 atoms in a molecule 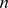 
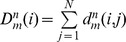
. The intermolecular similarity between molecules 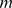 and 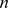 is given by the Tanimoto coefficient for a pairwise comparison of molecules [139]:
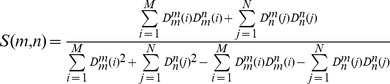



Lid conformations are grouped into clusters by comparing the intermolecular similarity coefficient. The first conformation is assigned to the first cluster. The next conformation is assigned to the cluster in which a cluster member has the highest similarity with the next molecule, if the similarity is above a threshold, chosen to be 0.85. Otherwise, the next conformation is assigned to a new cluster. The first member of the a cluster is called the cluster center. After all lid conformations are assigned to clusters, these conformations are arranged in new order, starting with the largest cluster and proceeding to the smallest cluster. The reordered set of lid conformations is subjected to the same clustering procedure.

## Supporting Information

Figure S1 **The Representative Snapshots from MD trajectory of MDM2-pS17: A Truncated Lid Model.** The representative snapshots from the 10 ns MD trajectory of the phosphorylated MDM2-pS17 presented at the time frames of 1 ns (**A**), 2 ns (**B**), 4 ns (**C**), 6 ns (**D**), 8 ns (**E**) and 10 ns (**F**). The MDM2 conformations are depicted in green ribbons.
(TIF)

Figure S2 **The Representative Snapshots from MD trajectory of MDM2-S17D: A Truncated Lid Model.** The representative snapshots from the 10 ns MD trajectory of the phosphomimetic MDM2-S17D presented at the time frames of 1 ns (**A**), 2 ns (**B**), 4 ns (**C**), 6 ns (**D**), 8 ns (**E**) and 10 ns (**F**). The MDM2 conformations are depicted in cyan ribbons.
(TIF)

Figure S3 **Equilibrium Conformational Ensembles of MDM2-pS17: A Comparison of the Truncated and Complete Lid.** (**A**)The representative lid conformations from the equilibrium ensembles of MDM2-pS17 obtained from simulations with the complete lid construct (colored in cyan) and the truncated lid (colored according to the B-factors values). (**B**) Structural superposition of the MDM2-pS17 lid conformations (shown in cyan, a complete lid model) with p53 (shown in blue sticks).
(TIF)

Figure S4 **Time-Dependent History of High Occupancy Salt Bridges.** The time-dependent history of salt bridges obtained from simulations of MDM2-pS17 (**A**) and MDM2-S17D (**B**). (**A**) The depicted high occupancy of the hydrogen bond interactions in MDM2-pS17 are between the following atoms : pS17(O2P)-K94(HZ2) in green; pS17(OT)-K94(HZ2) in blue and pS17()-K94(HZ2) in red. (**B**) The depicted high occupancy of the hydrogen bond interactions in the in the MDM2-pS17 is between S17D (O)-H96 (HE2).
(TIF)

Figure S5 **Structure-Functional Coupling of the Lid and Y100 Gating Dynamics.** The ensembles of the closed lid MDM2-pS17 conformations (**A**) and semi-closed MDM2-S17D conformations (**B**) are depicted using representative MDM2 conformations from 10 dominant clusters obtained from MD simulations. The Y100 residue is shown in “ball-and-stick” model according to the atom-based coloring scheme. While the closed conformations of the pS17 lid can induce the “in” conformation of Y100 (**A**), the semi-closed S17D conformations induce rotation of the gate-keeper residue towards the open (“out”) position (**B**). A ribbon-based representation of the MDM2 conformational ensembles was used. Coloring is according to the B-factors values (blue-to-red spectrum) reflecting protein nobilities of the MDM2 residues (from more rigid-blue regions to more flexible-red regions).
(TIF)

Figure S6 **Structural Analysis of the MDM2 Lid Ensembles: Modulation of the MDM2 Ligand Binding.** (**A**) Structural alignment of the closed lid structure from the equilibrium ensemble of the phosphorylated pS17 lid (in green ribbons) and the crystal structure of MDM2 with nutlin (in blue ribbons) [58]. The position of the lid is indicated with an arrow. (**B**) A close-up of the MDM2 binding site shows the overlap and severe interference between the closed lid (pointed to by an arrow) and nutlin (in blue sticks). P20 of the closed lid overlaps with the bromophenyl nutlin group and the nutlin ethyl ether side chain interferes with pS17 interactions. (**C**) Structural alignment of the semi-closed lid structure from the equilibrium ensemble of the phosphomimetic S17D lid (in green ribbons) and the crystal structure of MDM2 with nutlin (in blue ribbons). The position of the lid is indicated with an arrow. (**D**) A close-up of the MDM2 binding site reveals that there is no overlap between the S17D lid (pointed to by an arrow) and nutlin (in blue sticks). The S17D lid conformation allows the bromophenyl group and the ethyl ether side chain of nutlin to freely occupy their native positions.
(TIF)

Figure S7 **Structural Analysis of the Predicted pS17 Lid Conformations.** The two lowest energy binding modes of the phosphorylated pS17 lid (**A, C**) were obtained using a combination of multiple docking simulations and structural clustering of low energy state. The “|closed” form of the bound pS17 lid compared with p53 binding (PDB ID 1YCR). P53 peptide is shown in grey. L26, W23 and F19 of p53 are shown in pink and the phosphorylated pS17 lid is shown in “ball-and-stick” model. The Connolly surface was generated using the phosphorylated lid. Structural changes in the MDM2 receptor (residue 26–109) upon binding of the phosphorylated lid in the two lowest energy clusters (**B, D**) were compared with the MDM2 receptor in the complex with p53 (PDB ID 1YCR ) (shown in magenta). The phosphorylated lid is represented in the “ball-and-stick” model and p53 is shown in grey color.
(TIF)

Figure S8 **Structural Analysis of the Predicted S17D Conformations.** The two lowest energy binding modes of the phosphorylated S17D lid (**A, C**) were obtained using a combination of multiple docking simulations and structural clustering of low energy state. The “|closed” form of the bound S17D lid compared with p53 binding (PDB ID 1YCR). P53 peptide is shown in grey. L26, W23 and F19 of p53 are shown in pink and the phosphorylated pS17 lid is shown in “ball-and-stick” model. The Connolly surface was generated using the phosphorylated lid. Structural changes in the MDM2 receptor (residue 26–109) upon binding of the phosphorylated lid in the two lowest energy clusters (**B, D**) were compared with the MDM2 receptor in the complex with p53 (PDB ID 1YCR ) (shown in magenta). The phosphomimetic lid is represented in the “ball-and-stick” model and p53 is shown in grey color.
(TIF)
