# Supplementary figures and images for: Simulating Molecular Mechanisms of the MDM2-Mediated Regulatory Interactions: A Conformational Selection Model of the MDM2 Lid Dynamics
Source: PLoS One. 2012 Jul 16;7(7):e40897. doi: 10.1371/journal.pone.0040897 (PMC3397965; doi:10.1371/journal.pone.0040897)

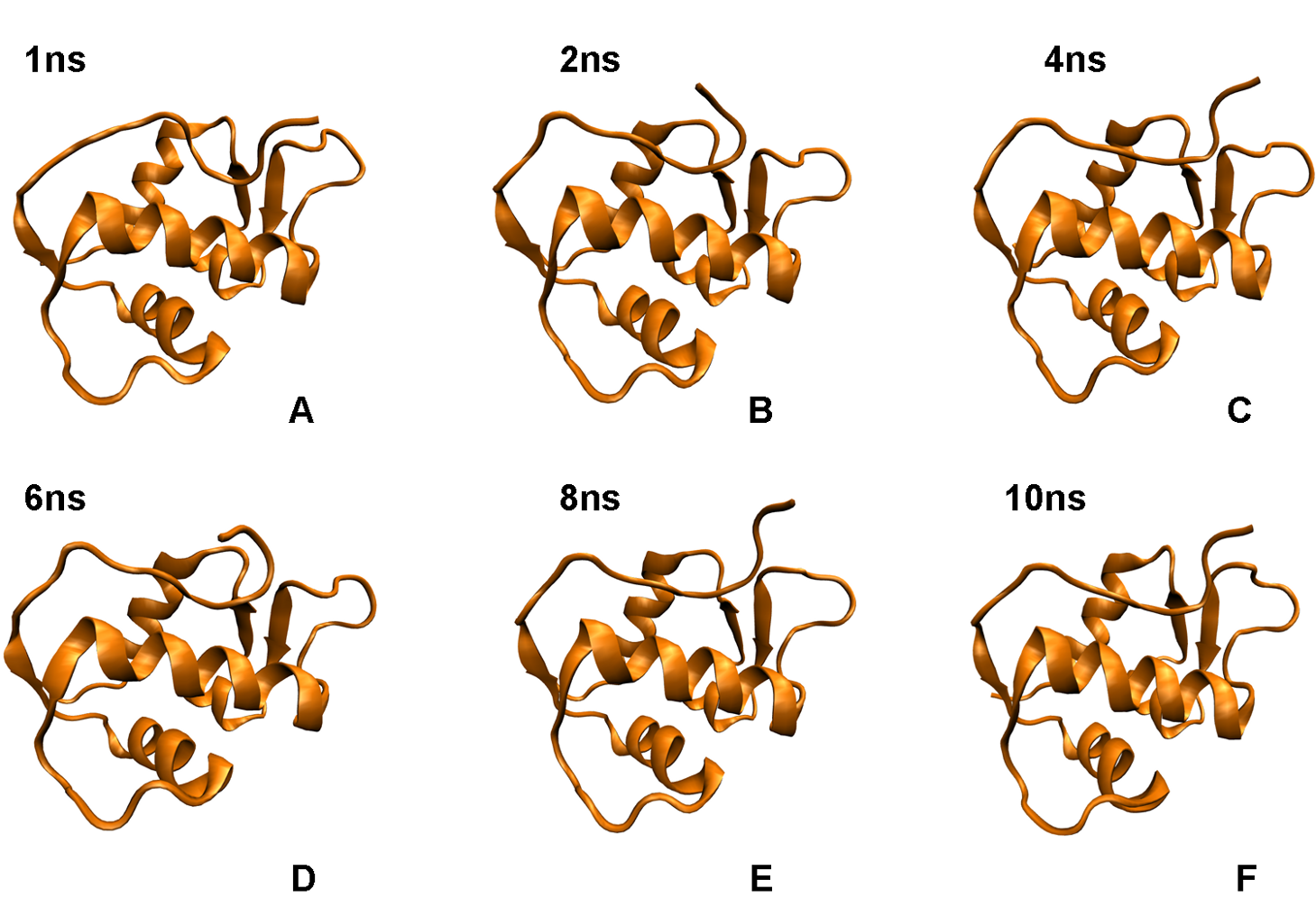

Supplement: Figure S1 — The Representative Snapshots from MD trajectory of MDM2-pS17: A Truncated Lid Model. The representative snapshots from the 10 ns MD trajectory of the phosphorylated MDM2-pS17 presented at the time frames of 1 ns (A), 2 ns (B), 4 ns (C), 6 ns (D), 8 ns (E) and 10 ns (F). The MDM2 conformations are depicted in green ribbons. (TIF) [file pone.0040897.s001.tif]

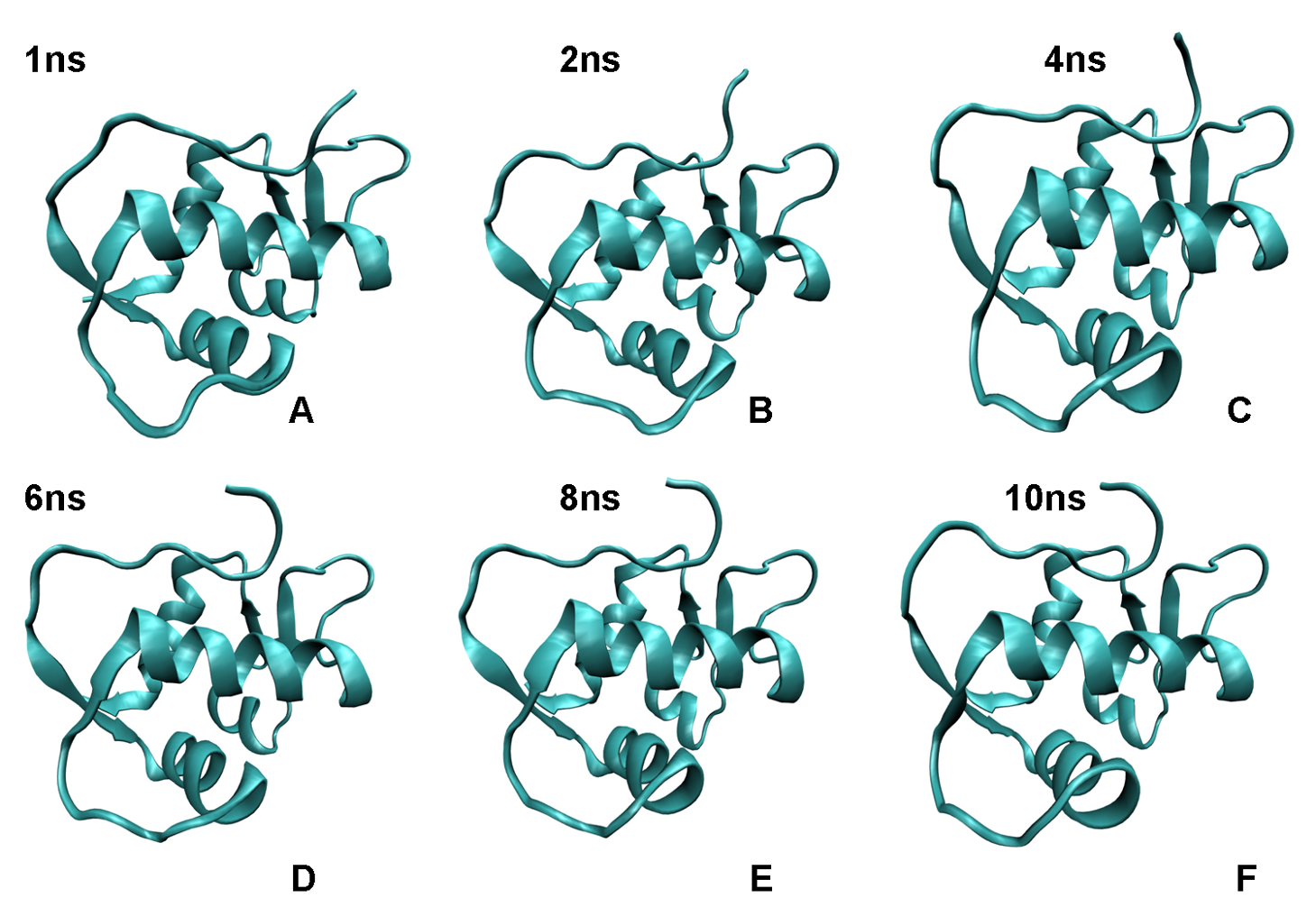

Supplement: Figure S2 — The Representative Snapshots from MD trajectory of MDM2-S17D: A Truncated Lid Model. The representative snapshots from the 10 ns MD trajectory of the phosphomimetic MDM2-S17D presented at the time frames of 1 ns (A), 2 ns (B), 4 ns (C), 6 ns (D), 8 ns (E) and 10 ns (F). The MDM2 conformations are depicted in cyan ribbons. (TIF) [file pone.0040897.s002.tif]

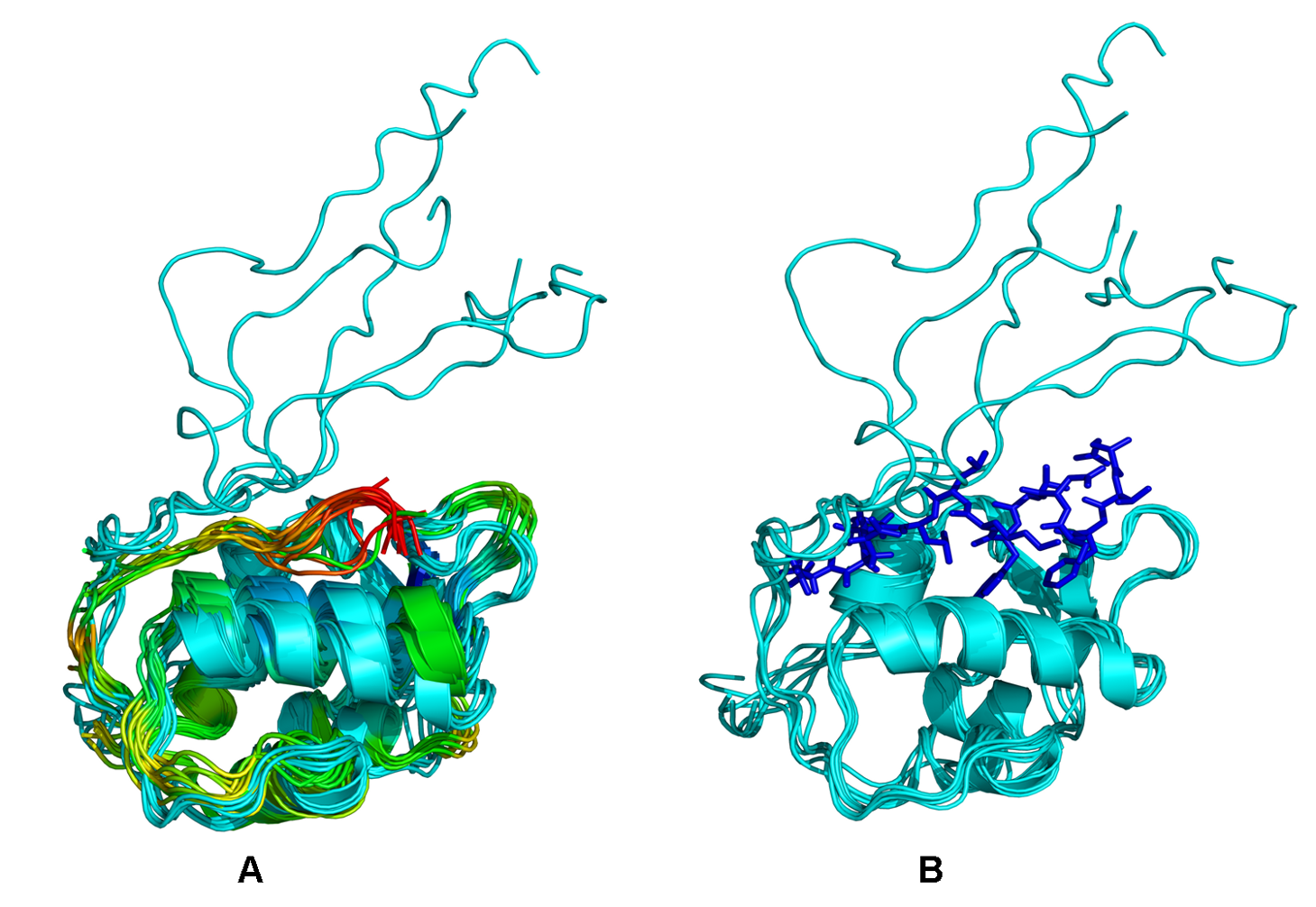

Supplement: Figure S3 — Equilibrium Conformational Ensembles of MDM2-pS17: A Comparison of the Truncated and Complete Lid. (A)The representative lid conformations from the equilibrium ensembles of MDM2-pS17 obtained from simulations with the complete lid construct (colored in cyan) and the truncated lid (colored according to the B-factors values). (B) Structural superposition of the MDM2-pS17 lid conformations (shown in cyan, a complete lid model) with p53 (shown in blue sticks). (TIF) [file pone.0040897.s003.tif]

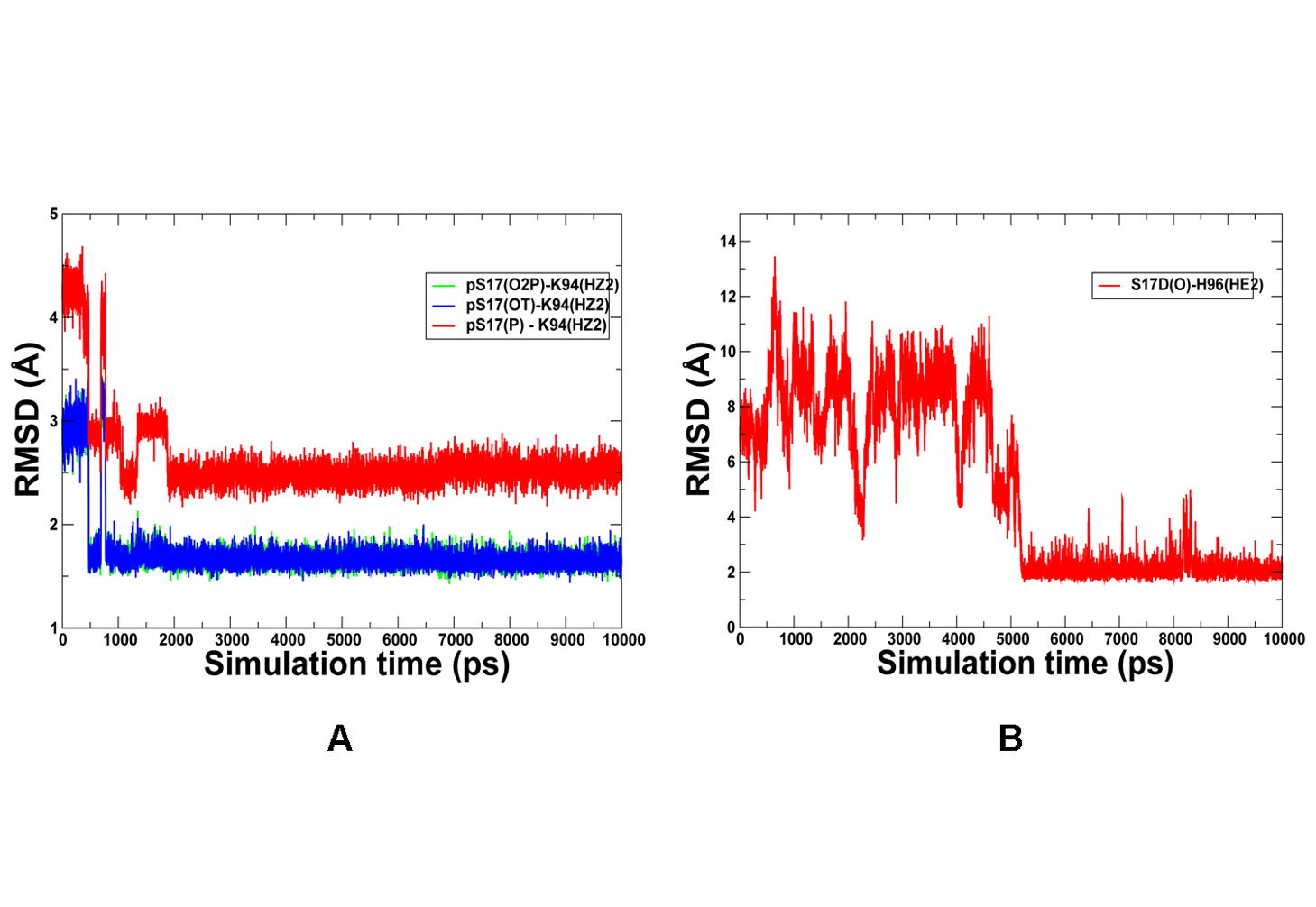

Supplement: Figure S4 — Time-Dependent History of High Occupancy Salt Bridges. The time-dependent history of salt bridges obtained from simulations of MDM2-pS17 (A) and MDM2-S17D (B). (A) The depicted high occupancy of the hydrogen bond interactions in MDM2-pS17 are between the following atoms : pS17(O2P)-K94(HZ2) in green; pS17(OT)-K94(HZ2) in blue and pS17()-K94(HZ2) in red. (B) The depicted high occupancy of the hydrogen bond interactions in the in the MDM2-pS17 is between S17D (O)-H96 (HE2). (TIF) [file pone.0040897.s004.tif]

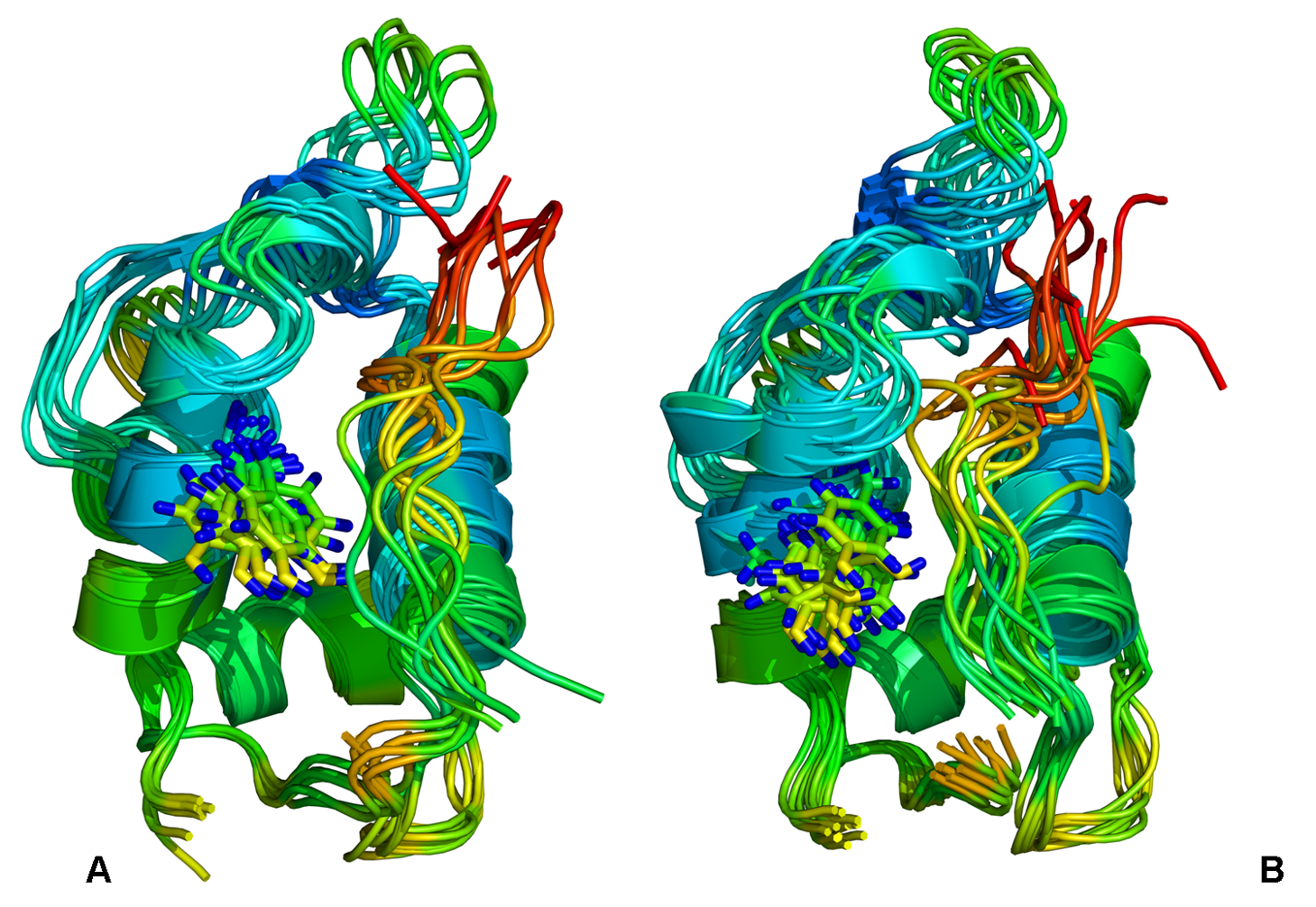

Supplement: Figure S5 — Structure-Functional Coupling of the Lid and Y100 Gating Dynamics. The ensembles of the closed lid MDM2-pS17 conformations (A) and semi-closed MDM2-S17D conformations (B) are depicted using representative MDM2 conformations from 10 dominant clusters obtained from MD simulations. The Y100 residue is shown in “ball-and-stick” model according to the atom-based coloring scheme. While the closed conformations of the pS17 lid can induce the “in” conformation of Y100 (A), the semi-closed S17D conformations induce rotation of the gate-keeper residue towards the open (“out”) position (B). A ribbon-based representation of the MDM2 conformational ensembles was used. Coloring is according to the B-factors values (blue-to-red spectrum) reflecting protein nobilities of the MDM2 residues (from more rigid-blue regions to more flexible-red regions). (TIF) [file pone.0040897.s005.tif]

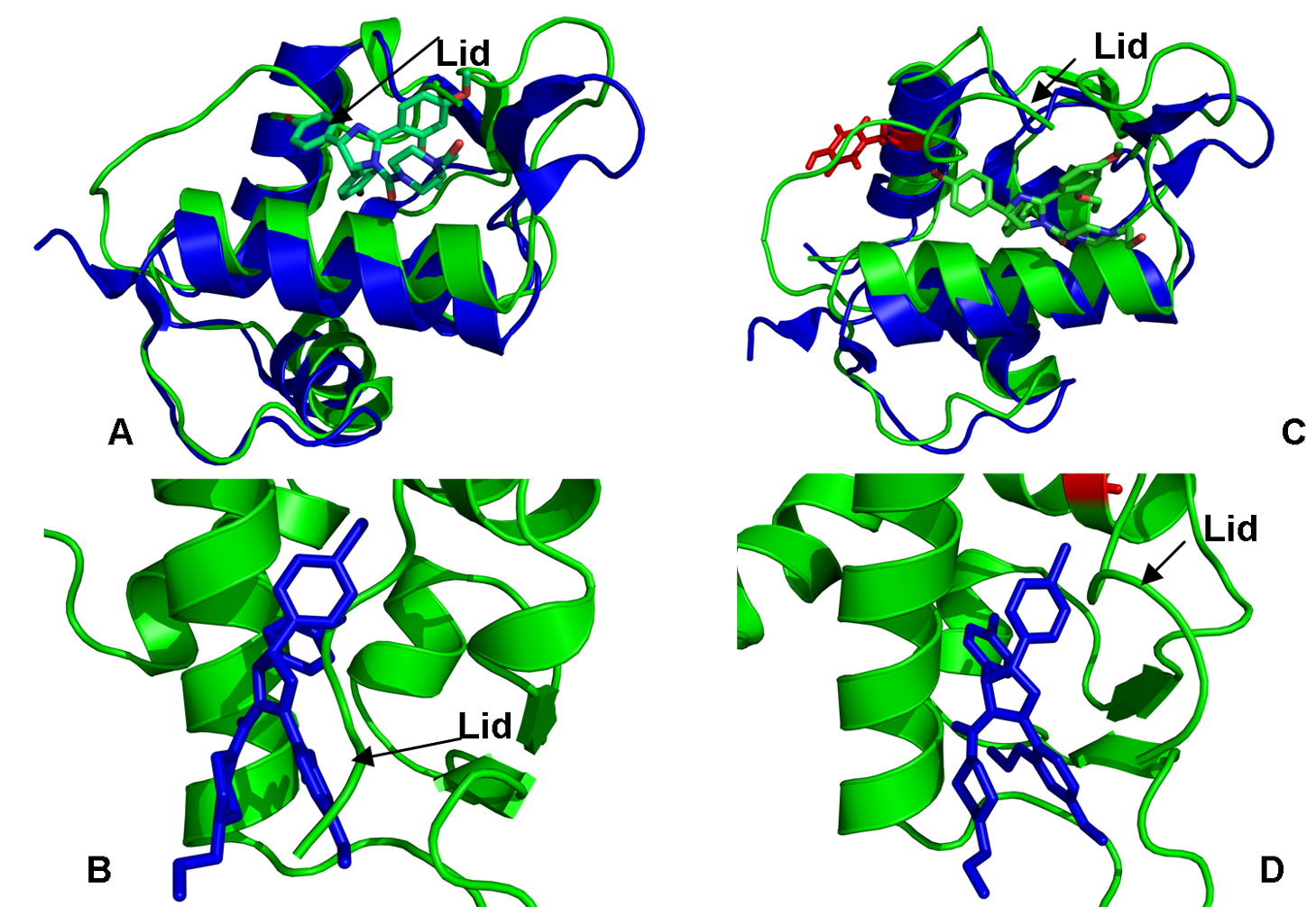

Supplement: Figure S6 — Structural Analysis of the MDM2 Lid Ensembles: Modulation of the MDM2 Ligand Binding. (A) Structural alignment of the closed lid structure from the equilibrium ensemble of the phosphorylated pS17 lid (in green ribbons) and the crystal structure of MDM2 with nutlin (in blue ribbons) [58]. The position of the lid is indicated with an arrow. (B) A close-up of the MDM2 binding site shows the overlap and severe interference between the closed lid (pointed to by an arrow) and nutlin (in blue sticks). P20 of the closed lid overlaps with the bromophenyl nutlin group and the nutlin ethyl ether side chain interferes with pS17 interactions. (C) Structural alignment of the semi-closed lid structure from the equilibrium ensemble of the phosphomimetic S17D lid (in green ribbons) and the crystal structure of MDM2 with nutlin (in blue ribbons). The position of the lid is indicated with an arrow. (D) A close-up of the MDM2 binding site reveals that there is no overlap between the S17D lid (pointed to by an arrow) and nutlin (in blue sticks). The S17D lid conformation allows the bromophenyl group and the ethyl ether side chain of nutlin to freely occupy their native positions. (TIF) [file pone.0040897.s006.tif]

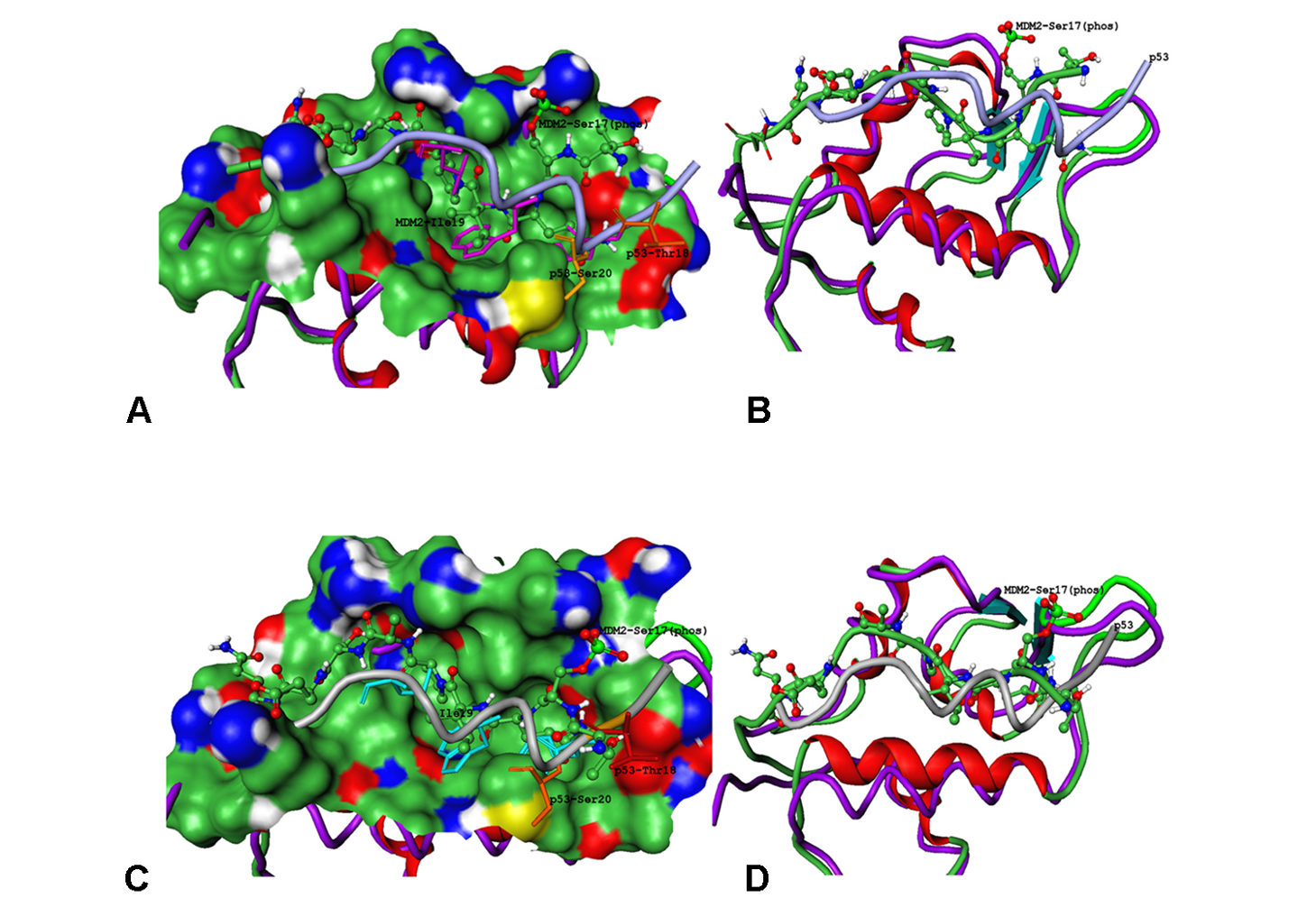

Supplement: Figure S7 — Structural Analysis of the Predicted pS17 Lid Conformations. The two lowest energy binding modes of the phosphorylated pS17 lid (A, C) were obtained using a combination of multiple docking simulations and structural clustering of low energy state. The “|closed” form of the bound pS17 lid compared with p53 binding (PDB ID 1YCR). P53 peptide is shown in grey. L26, W23 and F19 of p53 are shown in pink and the phosphorylated pS17 lid is shown in “ball-and-stick” model. The Connolly surface was generated using the phosphorylated lid. Structural changes in the MDM2 receptor (residue 26–109) upon binding of the phosphorylated lid in the two lowest energy clusters (B, D) were compared with the MDM2 receptor in the complex with p53 (PDB ID 1YCR ) (shown in magenta). The phosphorylated lid is represented in the “ball-and-stick” model and p53 is shown in grey color. (TIF) [file pone.0040897.s007.tif]

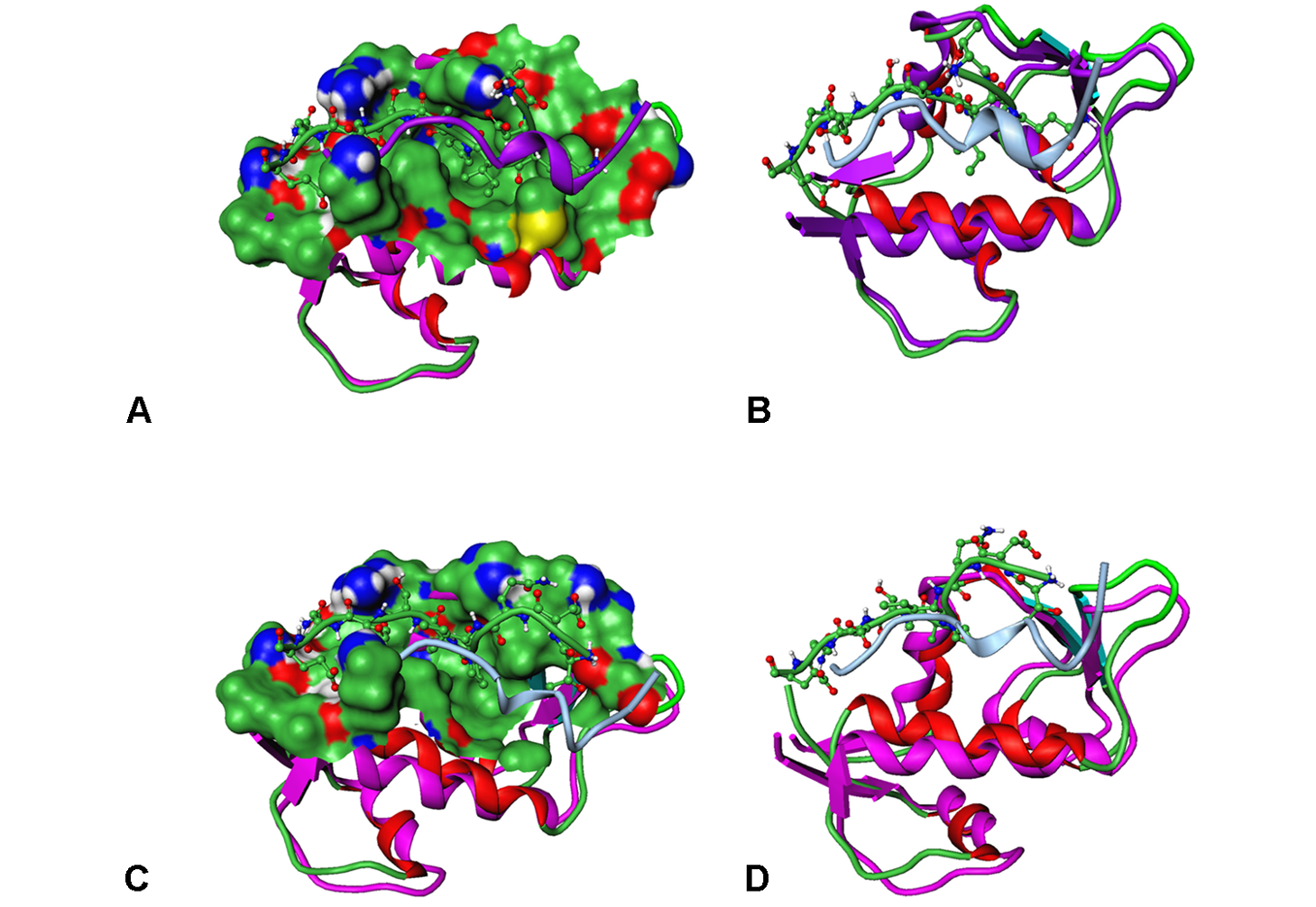

Supplement: Figure S8 — Structural Analysis of the Predicted S17D Conformations. The two lowest energy binding modes of the phosphorylated S17D lid (A, C) were obtained using a combination of multiple docking simulations and structural clustering of low energy state. The “|closed” form of the bound S17D lid compared with p53 binding (PDB ID 1YCR). P53 peptide is shown in grey. L26, W23 and F19 of p53 are shown in pink and the phosphorylated pS17 lid is shown in “ball-and-stick” model. The Connolly surface was generated using the phosphorylated lid. Structural changes in the MDM2 receptor (residue 26–109) upon binding of the phosphorylated lid in the two lowest energy clusters (B, D) were compared with the MDM2 receptor in the complex with p53 (PDB ID 1YCR ) (shown in magenta). The phosphomimetic lid is represented in the “ball-and-stick” model and p53 is shown in grey color. (TIF) [file pone.0040897.s008.tif]
